# Comparative transcriptional profiling identifies takeout as a gene that regulates life span

**DOI:** 10.18632/aging.100146

**Published:** 2010-05-11

**Authors:** Johannes Bauer, Michael Antosh, Chengyi Chang, Christoph Schorl, Santharam Kolli, Nicola Neretti, Stephen L. Helfand

**Affiliations:** ^1^ Department of Molecular Biology, Cell Biology and Biochemistry, Division of Biology and Medicine, Brown University, Providence, RI 02912, USA; ^2^ Institute for Brain and Neural Systems, Brown University, Providence, RI 02912, USA; ^3^ Department of Physics, Brown University, Providence, RI 02912, USA; ^4^ These authors shared equally in the work; ^5^ Present address: Department of Biological Sciences, Southern Methodist University, 6501 Airline Drive, 237-DLS, Dallas, TX 75275, USA

**Keywords:** Dietary restriction, Calorie restriction, microarrays, Drosophila melanogaster, Sir2, p53, Rpd3, Indy, methuselah (mth), chico, life span extension, and takeout

## Abstract

**A major challenge in translating the positive effects of dietary restriction (DR) for the improvement of human health is the development of therapeutic mimics.  One approach to finding DR mimics is based upon identification of the proximal effectors of DR life span extension. Whole genome profiling of DR in *Drosophila* shows a large number of changes in gene expression, making it difficult to establish which changes are involved in life span determination as opposed to other unrelated physiological changes. We used comparative whole genome expression profiling to discover genes whose change in expression is shared between DR and two molecular genetic life span extending interventions related to DR, increased dSir2 and decreased Dmp53 activity. We find twenty-one genes shared among the three related life span extending interventions.  One of these genes, *takeout,* thought to be involved in circadian rhythms, feeding behavior and juvenile hormone binding is also increased in four other life span extending conditions: *Rpd3, Indy*, *chico* and *methuselah*.  We demonstrate *takeout* is involved in longevity determination by specifically increasing adult *takeout* expression and extending life span. These studies demonstrate the power of comparative whole genome transcriptional profiling for identifying specific downstream elements of the DR life span extending pathway.

## Results

### Genetic background affects the specific genes that respond to DR

**Figure 1.**
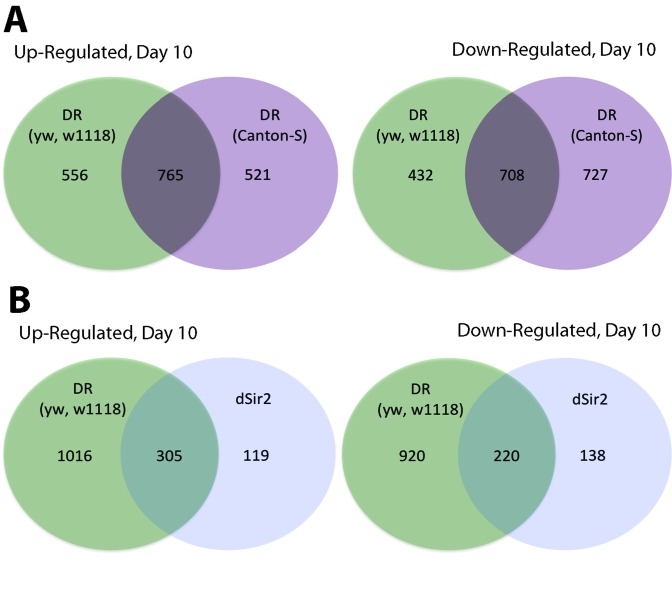
Comparison of genes upregulated and downregulated in yw/w ^1118^ DR, Canton-S CR and dSir2 overexpressed long-lived flies at Day 10. (**A**) Venn diagram comparing the upregulated and downregulated genes for DR flies in a yw/w^1118^ and a Canton-S background at age 10 days.  (**B**) Venn diagram comparing upregulated and downregulated genes in DR long-lived flies and dSir2 overexpressing long-lived flies at age 10 Days.  DR flies and dSir2 overexpressing flies are in an identical genetic background.  The majority of genes expressed in dSir2 overexpression are also expressed in DR (72% upregulated and 61% downregulated). Verification of microarray data using qPCR is in Supplementary Figure 3.

We examined the relative change in gene expression under DR conditions in whole female flies at Days 10 and 40 using flies from a combined inbred *yw*/*w^1118^* background and a Canton-S background.  The DR conditions used (1.5N high calorie and 0.5N low calorie; [1]) extend life span by 30-40% in both of these backgrounds.  Employing criteria of > 1.5 fold change and < 0.01 p value we found that the DR flies in the *yw*/*w^1118^* background showed 1321 genes increased at Day 10 and 1140 genes decreased at Day 10 (Figure 1A).  At Day 40 the *yw*/*w^1118^* CR flies had only 129 genes increased and 19 genes decreased (Supplementary Figure 1).  In the Canton-S background 1286 genes increased with DR at Day 10 and 1435 genes decreased with DR at Day 10 (Figure 1A).  At Day 40, 746 genes were increased and 715 genes were decreased in DR in the Canton-S background (Supplementary Figure 1).  Of the genes that increased or decreased in DR at Day 10 approximately 55-60% (765 up; 708 down) of them were shared between the two different fly backgrounds (*yw*/*w^1118 ^*and Canton-S). GOstat analysis of the genes altered by DR at day 10 and day 40 in these two different inbred genetic backgrounds revealed changes in biological functions similar to those previously described for DR in an outbred background of *Drosophila*[2-4]. (Supplementary Table 1).

These studies indicate that by day 10 there are a substantial number of genes expressed differentially by DR flies: 2461 in *yw*/*w^1118^* background; 2721 in Canton-S background and 1473 shared in both backgrounds. These changes should represent an inclusive set of most of the gene expression changes associated with DR including those unrelated to life span extension, but induced as a result of the nutritional challenge of DR. For example, in addition to extending life span in flies, DR also leads to a reduction in female fertility.  The decrease in fertility is not thought to be a primary component of the life span extending effect [5-7].

### Gene expression changes in dSir2 overexpressing long-lived flies overlaps with DR long-lived flies

In order to identify genes involved more specifically in DR life span extension we compared the changes in gene expression in DR with those induced by a specific molecular genetic life span extending intervention related to DR that does not cause a decrease in female fertility; an increase in adult neuronal dSir2 expression [6]. To improve the sensitivity in detecting shared changes in gene expression in DR and dSir2 overexpressing flies we compared these two interventions in genetically identical flies by using the inducible RU486 system [8,9].  A cohort of genetically identical flies possessing the GeneSwitch Elav driver (GSElav) and a construct permitting overexpression of dSir2 were randomly assigned to three different conditions: (i) high calorie food with EtOH diluent; (ii) low calorie food with EtOH diluent; and (iii) high calorie food with RU486.

A great deal of overlap in gene expression is seen between DR and neuronal specific dSir2 overexpression (Figure 1B). Of the 782 genes that change with neuronal specific dSir2 overexpression, 525 or 67% were shared with DR (72% upregulated and 61% downregulated).  When the comparison is made between dSir2 overexpression and the genetically less related Canton-S DR the overlap is only 55% (428 genes out of 782—Supplementary Figure 1).

Examination of the biological nature of the shared changes between DR and dSir2 life span extension at Day 10 using GOstat shows 148 shared categories decreasing and 72 shared categories increasing (a category contains at least 5 genes and a GOstat P value <0.05).  The dSir2 long-lived flies share 78% of their downregulated and 72% of their upregulated GO categories with DR (Supplementary Table 1).  The comparison between DR and dSir2 overexpression also confirms the phenotypic observation that female reproduction is more significantly affected in DR than in dSir2 overexpression. DR downregulates 31 GO categories related to female reproduction, while dSir2 overexpression downregulates only 11 GO categories related to female reproduction (Supplementary Table 1).

**Figure 2.**
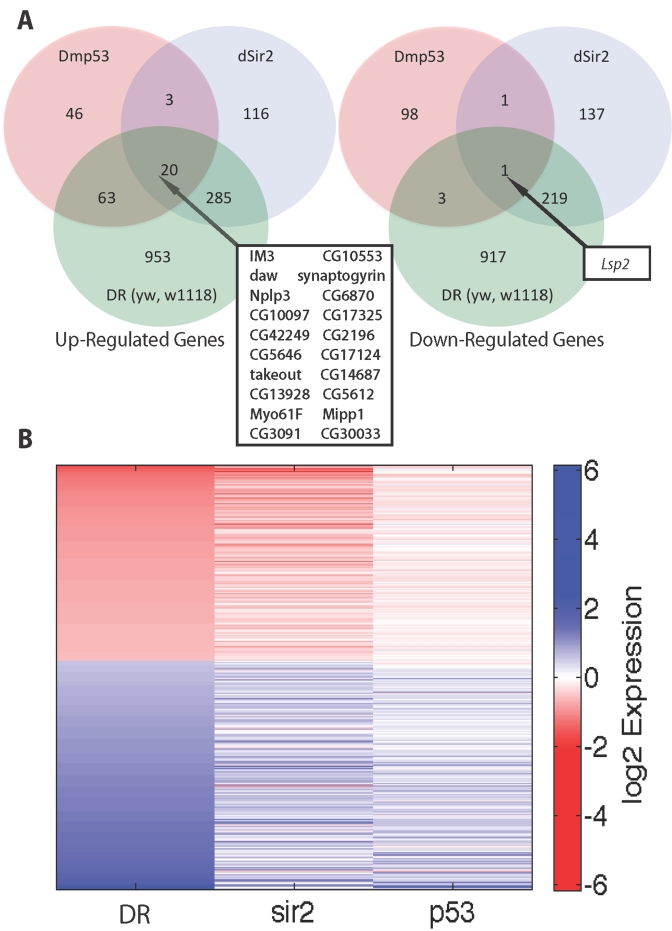
Comparison of genes upregulated and downregulated in DR, dSir2 overexpression and DN-Dmp53 expressing long-lived flies at Day 10. (**A**) Venn diagrams comparing upregulated and downregulated genes in DR, dSir2 overexpression, and DN-Dmp53 in a yw/w^1118^ background at age 10 Days. Genes intersecting in all 3 sets are noted in box with arrow. (**B**) Heatmap comparing the average log2 fold changes for genes significantly altered in the yw/w^1118^ DR with the equivalent genes in dSir2 and DN-Dmp53 expressing flies.

### Gene expression changes in DN-Dmp53 expressing long-lived flies overlaps with DR and dSir2 overexpressing long-lived flies

  Since a reduction in Dmp53 activity is a downstream component of the DR/Sir2 life span extending pathway [10,11] we compared the changes in gene expression of DN-Dmp53 long-lived flies to DR and dSir2 overexpression in a similar genetic background. Examination of the changes in gene expression at Day 10 in flies expressing DN-Dmp53 revealed 132 genes are upregulated and 103 genes are down regulated (Figure 2A).  Of the 235 genes that change with DN-Dmp53 expression, 87 or 37% were shared with DR (63% upregulated and 4% downregulated) and 88 or 37% were shared with dSir2 (65% upregulated and 2% downregulated) (Figure 2A).  The relationship between changes in gene expression between DR, dSir2 and Dmp53 is illustrated by the heat map in Figure 2B. Only one shared gene is seen at Day 40 (Supplementary Figure 1.) All but one of the 7 GO categories upregulated in the DN-Dmp53 expressing flies (endopeptidases, serine-type peptidase, serine-hydrolase, serine-type endopeptidase, peptidase, and defense response) are found in the upregulated GO categories of dSir2 and DR, while none of 15 GO categories downregulated in the DN-Dmp53 expressing flies are seen with dSir2 or DR (Supplementary Table 1).  Consistent with the normal fertility of the DN-Dmp53 expressing long-lived flies [5] we found no GO categories related to decreased female reproduction in the DN-Dmp53 expressing flies (Supplementary Table 1).

**Figure 3.**
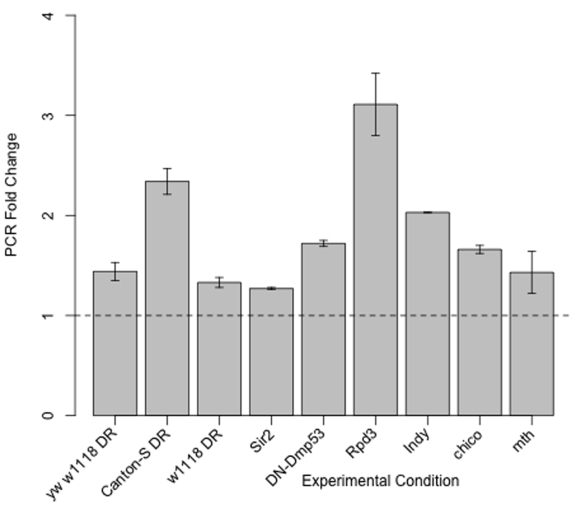
*takeout* mRNA expression is increased in *yw/w^1118^* DR, Canton-S DR, w^1118^ DR, *Sir2* overexpression, DN-Dmp53 expression, *Indy*, *Rpd3*, *methuselah* (*mth*) and *chico*. Fold change increase by qPCR of takeout mRNA from 10-Day old flies from these twelve life span extending conditions as compared to their genetically or dietary matched controls.

### Comparative whole genome expression profiling of DR, dSir2 and Dmp53 reveals a small set of shared genes

 Comparison of the specific genes shared at Day 10 between these three related life span extending interventions (DR, dSir2 expression and DN-Dmp53 expression) show 20 genes upregulated and 1 gene down regulated (Figure 2A). Among the 20 upregulated genes are  four genes associated with chromatin  structure or maintenance (CG42249, CG5612, CG17325, CG4123), three genes associated with circadian rhythm (CG10553, CG13928 and *takeout*), two genes involved in neural activity (*Nplp3-neuropeptide-like precursor 3*, *synaptogyrin*), two genes involved in detoxification/chaperone activity (CG3091, CG6870), two genes involved in muscle maintenance (Myo61F, CG14687) and genes related to immune function (IM3-induced immune molecule 3), growth factor activity (*dawdle*-activin), and feeding behavior and response to starvation (*takeout*) [12].  The single downregulated gene is *Lsp2 *(*larval serum protein-2*).

### *takeout* is upregulated in other life span extending interventions

Of the 21 genes shared among the DR, dSir2 and DN-Dmp53 long-lived flies, *takeout* was the only gene significantly altered in transcriptional profiles of *Indy* long-lived flies [13]. We confirmed *takeout* was increased in *Indy* long-lived mutants by qPCR and found *takeout* to be increased in *Rpd3*, *chico*, and*methuselah *mutants*,* single gene mutations that extend life span [14-16] (Figure 3). *takeout* was also found to be upregulated in DR in the Canton-S background and in an independent *w^1118^* background by qPCR.

### Increasing*takeout* expression extends life span

**Figure 4.**
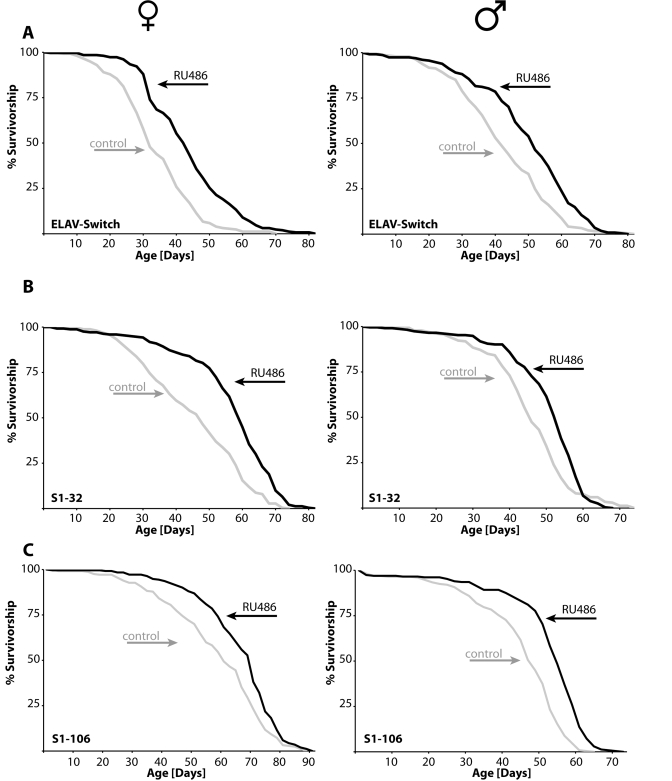
** Overexpression of *takeout*in either of three different adult tissues extends life span of males and females**.(**A**) Expression of *takeout* in the adult nervous system using the ELAV-Switch neuronal specific GAL4 driver leads to ~25% increase in mean longevity. (**B**) Flies expressing *takeout* in the head fat body, S1-32 pericerebral fat body specific GAL4 driver, have ~20% extension of mean life span, while *takeout* expression in the abdominal fat body, S1-106 abdominal fat body specific GAL4 driver, (**C**) extends fly life span by ~12-18% (females: left panel; males: right panel; statistical analysis in Table 1 and 2; grey: controls; black: *takeout*).

Given the association between the known phenotypes of *takeout* and longevity determination (feeding behavior, response to starvation and juvenile hormone binding properties; [17-23]) and our finding of *takeout's* upregulation in a number of different life span extending conditions, we examined the effect on life span of selectively increasing *takeout*.  We found overexpression of *takeout* in adult neurons, pericerbral fat body or abdominal fat body extends male and female life span (Figure 4, Tables 1 and 2).

**Table 1.** The effect of *takeout* expression on female life span.

**Driver**	**Mean LS (vs.ctrl)**	**Mean LS extension**	**Median LS (vs. ctrl)**	**Median LS extension**	**Max LS (vs. ctrl)**	**Max LS extension**	**Number of flies (control;experimental)**	**χ2 **	**p-value**
ELAV Switch	48/44	9%	48/44	9%	64/60	7%	275 248	12.92	0.0003
ELAV Switch	43/34	26%	44/32	38%	64/52	23%	255 257	71.45	<0.0001
S_1_-32	57/47	21%	60/48	25%	74/68	9%	252 243	71.23	<0.0001
S_1_-32	51/48	6%	54/50	8%	69/68	1%	248 245	8.994	0.0027
S_1_-106	65/58	12%	70/60	17%	82/80	3%	247 252	21.44	<0.0001
da	50/40	25%	50/40	25%	66/56	18%	256 247	119.3	<0.0001

**Table 2.** The effect of takeout expression on male life span.

**Driver**	**Mean LS (vs.ctrl)**	**Mean LS extension**	**Median LS (vs. ctrl)**	**Median LS extension**	**Max LS (vs. ctrl)**	**Max LS extension**	**Number of flies (control;experimental)**	**χ2 **	**p-value**
ELAV Switch	53/52	2%	56/54	4%	66/64	3%	228 190	1.34	0.247
ELAV Switch	50/43	16%	52/42	24%	70/62	13%	241 228	31.08	<0.0001
S_1_-32	50/46	9%	54/46	17%	66/60	10%	234 234	18.34	<0.0001
S_1_-32	66/63	5%	66/64	3%	84/84	0%	247 243	1.669	0.1964
S_1_-106	52/44	18%	54/48	13%	64/60	7%	227 233	81.9	<0.0001
da	53/43	23%	58/42	38%	64/64	0%	246 252	37.6	<0.0001

### Long-lived*takeout* overexpressing flies upregulate a subset of the genes upregulated in DR, dSir2 and DN-Dmp53 long-lived flies

**Figure 5.**
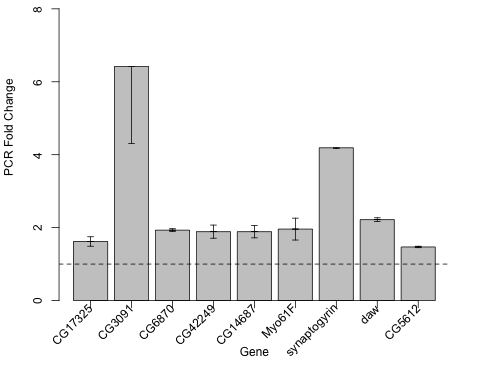
Long-lived *takeout* overexpressing flies have increased expression of a subset of the upregulated genes found in DR, dSir2 overexpression and DN-Dmp53 expressing flies. Overexpression of *takeout* in adult neurons increases the expression of a subset of 9 genes from the 19 upregulated genes shared in *yw/w^1118^* DR, dSir2 overexpression and DN-Dmp53 expressing long-lived flies at Day 10.  Fold change increase by qPCR of each of the noted genes using mRNA extracted from 10-Day old flies induced to express *takeout* (Elav-GeneSwitch-UAS-to) as compared to genetically identical controls Elav-GeneSwitch; UAS-to flies without fed diluent.

It is our hypothesis that the twenty genes upregulated in flies whose life span is extended by DR, dSir2 or DN-Dmp53 may represent elements downstream in the DR life span extending pathway.  Demonstration that upregulation of *takeout* results in life span extension confirms that *takeout* is a likely component of the DR life span extending pathway.  As a first step in identifying ad-ditional downstream genes associated with DR life span extending pathways we examined which of the 19 re-maining upregulated genes are also upregulated in long-lived  *takeout* expressing  flies using qPCR on-  mRNA from the flies overexpressing *takeout* in adult neurons. Nine out of the 19 genes showed a greater than 1.4 fold increase in expression in the *takeout* overexpressing long-lived flies (Figure 5). These include: (i) *dawdle*, a homologue of activin, coding for a transforming growth factor beta receptor binding protein; (ii) CG6870 coding for the cytochrome B5 detoxifying enzyme; (iii) CG3091, a gene coding for cellular retinaldehyde-binding/alpha-tocopherol transport that may be involved in detoxification; (iv) CG17325, a gene whose product interacts with chromatin related proteins such as SNR1; (v) CG42249, a gene with a predicted polycomb/trithorax response element; (vi) CG14687, with Myosin light chain binding properties; (vii) *Myo61F*; (ix) *synaptogyrin*; and (ix) CG5612 function unknown [12].

## Discussion

Examination of the changes in gene transcription profiles for DR in two different genetic backgrounds reveals the presence of a shared set of genes suggesting that one or more conserved core longevity-signaling pathways may exist to regulate lifespan in response to nutrient conditions. Such core longevity-signaling pathways may be utilized by other life span extending interventions not directly related to DR, and could help explain some of the cross-talk seen between DR and alterations in insulin/insulin-like signaling.

The set of common DR induced genes found represents genes important in life span extension as well as genes associated with other nutrient induced physiological functions not directly related to life span, such as decreased fertility.  A comparative approach can be used to enrich for genes more specifically related to life span extension by examining life span extending interventions related to DR that do not have some of the same untoward effects as DR.  Expression of dSir2 and DN-Dmp53 are two life span extending interventions that are part of the DR life span extending pathway in flies, but do not have decreased fertility [5,6].  The whole genome expression profiles of flies on a DR diet and long-lived dSir2 expressing flies on a normal diet show a substantial overlap in changes in gene expression, supporting the observations linking dSir2 and DR (Figure 1B).  As predicted, while DR has many GO categories associated with downregulation of fertility [31], fewer are seen with dSir2 long-lived flies [11] and none in DN-Dmp53 expressing long-lived flies (Supplementary Table 1).

Comparisons of whole genome profiles of flies on DR, expressing dSir2 and expressing DN-Dmp53 revealed a small set of 21 commonly genes predicted to be enriched for genes involved in longevity regulation (Figure 2A).  *takeout* (*to*), was selected to be further examined based upon *takeout's* known role in regulating feeding behavior and the starvation response [17-23] as well as its presence in a set of upregulated genes from transcriptional profiles of another life span extending mutant in the fly,* Indy*[13]. Examination of *takeout* mRNA levels showed that in addition to *takeout* being upregulated in DR from three different fly backgrounds it is also upregulated in four additional separate life span extending mutants *chico*,*Rpd3*, *methuselah* and *Indy*[14-16,24] (Figure 3).  Confirmation of *takeout*'*s* role in longevity determination was demonstrated by overexpression in the fat body or nervous system of adult flies and extending life span (Figure 4).

The level of expression of *takeout* in the overexpression studies is similar to the induction seen with DR (Figures 3 and S2), however lifespan extension by *takeout* over-expression is less than what is observed with DR. This effect may be due to the *w^1118^* background used in these particular experiments, which is known to have a reduced DR response compared to other backgrounds [25].  Alternatively, *takeout* may be only one of several genes in the DR life span extending pathway that can positively influence lifespan. Other genes, including the additional 19 upregulated genes identified through comparative transcriptional profiling may increase lifespan incrementally, adding up to the lifespan extension total seen in DR or through other genetic interventions.

The mechanism by which increased *to* expression leads to life span extension is not known. Interestingly, *takeout* is regulated in a circadian fashion [18,19,26]. Increasingly, the link between the circadian system, food intake and aging has been observed [27]. The finding that expression of *takeout* from any of three different tissues (adult neurons, pericerebral fat body, abdominal fat body) extends life span suggests that the life span related functions of *takeout* could be due to its hypothesized function as a secreted Juvenile Hormone (JH) binding protein [17,20,22]. Although it is not known if the JH binding domain of *takeout* is functional, reduction of JH levels have been linked to increased longevity in grasshoppers [28]. *takeout* may bind JH in the hemolymph, thereby reducing JH bioavailability. It has been speculated that the insect ecdysone-JH system may be the functional equivalent of the mammalian thyroid hormone-prolactin axis, which controls important aspects of mammalian basal metabolism [29,30]. Therefore, proteins such as *takeout* may be important mediators, linking a nutrient sensing network (DR, dSir2, insulin/insulin-like signaling) with an effector network (JH signaling), which in turn controls behavioral and physiological adaptation pathways.

Our data suggest that multi-factorial gene expression profiling can be successfully used to enrich for genes directly involved in the regulation of longevity, filtering out the noise of other physiological processes. Further refinement of this unbiased approach will be invaluable for discovering factors and signaling pathways involved in aging and lifespan regulation by a variety of modalities and for the identification of targets for specific therapeutic interventions.

## Experimental procedures

All flies were kept in a humidified (50%), temperature-controlled incubator with 12 hour on/off light cycle at 25˚C in vials containing standard cornmeal medium [6]. The ELAV-GeneSwitch line was from H. Keshishian (Yale University, New Haven, CT), S1-32-GeneSwitch, S1-106-GeneSwitch, *chico* and matched genetic controls for *chico* were from M. Tatar (Brown University, Providence, RI), *methuselah* and matched genetic controls from W. Ja (Caltech, Pasadena, CA) and UAS-*takeout*(UAS-*to*) was from B. Dauwalder (University of Houston, Houston, TX). All other lines (except *Indy*) were from the Bloomington *Drosophila* Stockcenter at Indiana University (Bloomington, IN).

*The following crosses and experimental treatments were used in the microarray and lifespan analyses: yw; ELAV-Geneswitch x P{EP}dSir2^EP2300^/CyO (Bloomington 24859) => ELAV-Geneswitch-dSir2^EP2300^ (-/+ RU486)                                     x P{GUS}-Dmp53^259H^/TM6 (Bloomington 6582)                                     => ELAV-Geneswitch- P{GUS}-Dmp53^259H^ (-/+ RU486) yw; S_1_-32/CyO               x UAS-to                                     => S_1_-32-UAS-to (-/+ RU486) yw; S1-106                    x UAS-to                                     => S1-106 -UAS-to (-/+ RU486)*

Life span analysis. Flies were collected under light anesthesia, randomly divided into treatment groups and housed at a density of 25 males and 25 females each per vial. At least ten such vials were used per treatment as per [31]. Flies were passed every other day and the number of dead flies recorded.

All life span experiments were performed on regular cornmeal food, and for induction with the GeneSwitch system, RU486 (Sigma) was added directly to the food to a final concentration of 200μM. The same concentration of diluent (EtOH) was added to control food. RU486 was administered from the day of eclosion. For expression with constitutive da-GAL4 driver, UAS-*takeout* was backcrossed to *w^1118^* for 10 generations and isogenic controls were generated from the last backcross. Statistical analyses, including log rank tests, were performed using the Prism suit of biostatistical software (GraphPad, San Diego). Maximum life span was calculated as the median age of the last surviving 10% of the population.

Microarrays. For microarray experiments of DR animals, Canton-S and a mixed yw/w^1118^ (the diluent controls from the genetic interventions below) line were aged for 10 or 40 days either on 1.5N or 0.5N food (15% sucrose and 15 yeast extract, or 5% sucrose and 5% yeast extract (all w/v), respectively) [1]. For genetic interventions, ELAV-GeneSwitch-dSir2^EP2300^ and ELAV-GeneSwitch-DN-Dmp53^259H^ flies were aged for 10 or 40 Days as described for the life span experiments on food containing diluent or RU486. Total RNA was isolated from at least 75 females using Trizol (Invitrogen) and further purified using RNeasy columns (QIAGEN). 5 μg total RNA was used with Affymetrix One Cycle DNA conversion Kit (Cat # 900431) and all steps were carried out according to the Affymetrix manual. Briefly, first RNA was converted to double stranded cDNA followed by a clean-up step using spin columns. The double stranded cDNA was amplified in an in-vitro transcription reaction overnight at 37 °C using Affymetrix IVT labeling kit (cat # 900449), resulting in biotin labeled cRNA. After clean-up of the labeled cRNA with spin columns, 15 μg of cRNA were fragmented using metal induced hydrolysis. 10 μg of the fragmented RNA were hybridized to Drosophila 2.0 arrays overnight at 45 °C, 60 rpm. The array was stained using Affymetrix Hybridization-Wash-Stain kit and Fluidics Script FS450_0002 on the Affymetrix 450 fluidics station and finally, the arrays were scanned using an Affymetrix 3000 G7 scanner. At least three independent biological replicates per intervention were analyzed.

Pre-processing of microarray data : The data was quantile normalized and summarized using GCRMA [32] to obtain expression scores in the log2 scale. A probeset was considered absent if its mean expression level was below the 25^th^ percentile (compared to the rest of the mean expressions for that condition) in both experiment and control. Absent probesets were removed from further analysis.

Differential Expression. A set of three biological replicates from both the treatment and control cohorts was used to identify differentially expressed probesets. Probesets with a p value (two sided t test) smaller than 0.01 and a fold change larger than 1.5 or smaller than 1/1.5 were selected as differentially expressed. These thresholds were chosen to minimize the number of false positives and false negatives in a comparison test of the microarray data of a pool of genes with PCR data from the same samples (Supplemental Figure 1). Probesets have been collapsed to genes after statistical selection for differential expression.

GOstat. The genes were analyzed using GOstat [4], which determines which sets of genes (called gene ontologies) are enriched in a list of genes. The input to GOstat is the list of differentially expressed genes for an experiment versus control comparison. For each gene ontology the intersection is found between the input list and the list of genes in the gene ontology. A p value is computed as the probability of obtaining an intersection at least as large as the one observed by random sampling using the hypergeometric distribution. p values were adjusted for multiple testing using Benjamini and Hochberg's False Discovery Rate algorithm [33]. Ontologies with an adjusted p value < 0.05 were considered as overrepresented.  Only gene ontologies containing at least 5 genes were considered.

Quantitative PCR. Total mRNA was isolated from at least 75 heads of 10-day old females using Trizol (Invitrogen) and further purified using the RNeasy kit (Qiagen). cDNA was generated with 0.5μg total mRNA in a 10μl reaction using the iScript cDNA synthesis kit (Bio-Rad). 0.8μl of the iScript reaction was used as qPCR template. qPCR was performed as described [10] on an ABI 7500 Real-Time PCR machine using the ABI SYBR-Green PCR master mix following the manufacturers instructions. Each qPCR reaction was performed using four biological replicates in triplicate each and normalized to mRNA from GAPDH or tubulin.

## Supplementary data

Supplementary Table 1 Gene Ontologies Up-regulated in yw, w1118 DR.

Supplementary Figure 1 Comparison of genes upregulated and downregulated in: yw/w ^1118^ Dr, Canton-S background at age 40 Days. (**B**) Venn diagram comparing upregulated and downregulated genes in DR long-lived flies and dSir2 overexpressing long-lived flies at age 40 Days.  DR flies and dSir2 overexpressing flies are in an identical genetic background. Canton-S DR and dSir2 overexpressed long-lived flies at Day 10 and Day 40. Venn diagram comparing the upregulated and downregulated genes for DR flies in a Canton-S background and dSir2 overexpressing long-lived flies at age 10 Days (**C**) and age 40 Days (**D**).  Canton-S DR flies and dSir2 overexpressing flies are in different genetic backgrounds. DR, dSir2 overexpression and DN-Dmp53 expressing long-lived flies at Day 40. (**E**) Venn diagrams comparing upregulated and downregulated genes in DR, dSir2 overexpression, and DN-Dmp53 in a yw/w^1118^  background at age 40 Days.

Supplementary Figure 2 *takeout* mRNA expression is increased in Elav GeneSwitch;UAS-to, S1-32; UAS-to and S1-106; UAS-to. Fold change increase by qPCR of *takeout* mRNA from 10-Day old flies from these three life span extending conditions as compared to their genetically matched controls.

Supplementary Figure 3 Verification of microarray data using qPCR. Each point on the graph represents a gene measured by both microarray and qPCR. The axes describe the fold change and p value of the microarray data. The red dots represent genes with a significant fold change (>20%) in PCR, and the blue dots represent genes with a non-significant fold change in PCR. The dotted lines define a box of the region where the PCR data is most likely to be significant--fold change > 1.5 (0.58 in log2 space) and p value < 0.01.
