# Supplementary material for: Comparative transcriptional profiling identifies takeout as a gene that regulates life span
Source: Aging (Albany NY). 2010 May 11;2(5):298–310. doi: 10.18632/aging.100146 (PMC2898020; doi:10.18632/aging.100146)
Supplement: Supplementary Table 1 [file aging-02-298-s001.pdf]

Table S1A: Gene Ontologies Up-regulated in yw, w1118 DR. All gene ontologies have at least 5 genes in the GO and p value < 0.05.

GO:0000038	very-long-chain fatty acid metabolic process
GO:0000122	negative regulation of transcription from RNA polymerase II promoter
GO:0000267	cell fraction
GO:0000270	peptidoglycan metabolic process
GO:0000902	cell morphogenesis
GO:0000904	cell development
GO:0001505	regulation of neurotransmitter levels
GO:0001704	formation of primary germ layer
GO:0001706	endoderm formation
GO:0001707	mesoderm formation
GO:0001708	cell fate commitment
GO:0001709	cell fate commitment
GO:0001710	mesodermal cell fate commitment
GO:0001736	establishment of planar polarity
GO:0001738	morphogenesis of a polarized epithelium
GO:0001751	compound eye photoreceptor cell differentiation
GO:0001752	compound eye photoreceptor fate commitment
GO:0001754	eye photoreceptor cell differentiation
GO:0001871	pattern binding
GO:0002117	larval development (sensu Amphibia)
GO:0002164	larval development
GO:0002165	instar larval or pupal development
GO:0002376	immune system process
GO:0003006	reproductive developmental process
GO:0003008	system process
GO:0003700	transcription factor activity
GO:0003702	RNA polymerase II transcription factor activity
GO:0003704	specific RNA polymerase II transcription factor activity
GO:0003779	actin binding
GO:0004035	alkaline phosphatase activity
GO:0004175	endopeptidase activity
GO:0004179	membrane alanyl aminopeptidase activity
GO:0004180	carboxypeptidase activity
GO:0004222	metalloendopeptidase activity
GO:0004252	serine-type endopeptidase activity
GO:0004263	chymotrypsin activity
GO:0004295	trypsin activity
GO:0004723	calcium-dependent protein serine/threonine phosphatase activity
GO:0005102	receptor binding
GO:0005179	hormone activity
GO:0005184	neuropeptide hormone activity
GO:0005200	structural constituent of cytoskeleton
GO:0005275	amine transmembrane transporter activity
GO:0005283	sodium:amino acid symporter activity
GO:0005326	neurotransmitter transporter activity
GO:0005328	neurotransmitter:sodium symporter activity
GO:0005342	organic acid transmembrane transporter activity
GO:0005416	cation:amino acid symporter activity
GO:0005509	calcium ion binding
GO:0005515	protein binding
GO:0005516	calmodulin binding
GO:0005549	odorant binding
GO:0005578	proteinaceous extracellular matrix
GO:0005604	basement membrane
GO:0005624	membrane fraction
GO:0005792	microsome
GO:0005859	muscle myosin complex
GO:0005865	sarcomere
GO:0005886	plasma membrane

GO:0005887	integral to plasma membrane
GO:0005911	intercellular junction
GO:0005912	adherens junction
GO:0005913	cell-cell adherens junction
GO:0006357	regulation of transcription from RNA polymerase II promoter
GO:0006508	proteolysis
GO:0006576	biogenic amine metabolic process
GO:0006582	melanin metabolic process
GO:0006629	lipid metabolic process
GO:0006810	transport
GO:0006811	ion transport
GO:0006812	cation transport
GO:0006817	phosphate transport
GO:0006820	anion transport
GO:0006874	cellular calcium ion homeostasis
GO:0006928	cell motility
GO:0006952	defense response
GO:0006955	immune response
GO:0006959	humoral immune response
GO:0007015	actin filament organization
GO:0007043	intercellular junction assembly
GO:0007154	cell communication
GO:0007155	cell adhesion
GO:0007164	establishment of tissue polarity
GO:0007165	signal transduction
GO:0007166	cell surface receptor linked signal transduction
GO:0007167	enzyme linked receptor protein signaling pathway
GO:0007169	transmembrane receptor protein tyrosine kinase signaling pathway
GO:0007186	G-protein coupled receptor protein signaling pathway
GO:0007200	G-protein signaling, coupled to IP3 second messenger (phospholipase C activating)
GO:0007218	neuropeptide signaling pathway
GO:0007242	intracellular signaling cascade
GO:0007267	cell-cell signaling
GO:0007268	synaptic transmission
GO:0007275	multicellular organismal development
GO:0007385	specification of segmental identity, abdomen
GO:0007399	nervous system development
GO:0007402	ganglion mother cell fate determination
GO:0007405	generation of neurons
GO:0007409	axonogenesis
GO:0007411	axonogenesis
GO:0007416	synaptogenesis
GO:0007417	central nervous system development
GO:0007419	ventral cord development
GO:0007422	nervous system development
GO:0007424	open tracheal system development
GO:0007427	epithelial cell migration, open tracheal system
GO:0007431	salivary gland development
GO:0007432	salivary gland boundary specification
GO:0007435	salivary gland morphogenesis
GO:0007439	ectodermal gut development
GO:0007444	imaginal disc development
GO:0007447	imaginal disc development
GO:0007450	dorsal/ventral pattern formation, imaginal disc
GO:0007451	dorsal/ventral pattern formation, imaginal disc
GO:0007472	wing disc morphogenesis
GO:0007473	wing disc proximal/distal pattern formation
GO:0007476	imaginal disc-derived wing morphogenesis
GO:0007478	leg disc morphogenesis
GO:0007480	imaginal disc-derived leg morphogenesis
GO:0007482	haltere development
GO:0007492	endoderm development

GO:0007494	midgut development
GO:0007498	mesoderm development
GO:0007500	mesodermal cell fate determination
GO:0007506	gonad development
GO:0007507	heart development
GO:0007517	muscle development
GO:0007519	skeletal muscle development
GO:0007525	somatic muscle development
GO:0007528	skeletal muscle fiber development
GO:0007548	sex differentiation
GO:0007552	metamorphosis
GO:0007600	sensory perception
GO:0007601	visual perception
GO:0007602	phototransduction
GO:0007619	courtship behavior
GO:0007622	rhythmic behavior
GO:0007623	circadian rhythm
GO:0007632	visual behavior
GO:0008020	G-protein coupled photoreceptor activity
GO:0008063	Toll signaling pathway
GO:0008083	growth factor activity
GO:0008084	imaginal disc growth factor activity
GO:0008092	cytoskeletal protein binding
GO:0008194	UDP-glycosyltransferase activity
GO:0008233	peptidase activity
GO:0008235	metalloexopeptidase activity
GO:0008236	serine-type peptidase activity
GO:0008237	metallopeptidase activity
GO:0008238	exopeptidase activity
GO:0008241	peptidyl-dipeptidase activity
GO:0008253	5′-nucleotidase activity
GO:0008271	secondary active sulfate transmembrane transporter activity
GO:0008277	regulation of G-protein coupled receptor protein signaling pathway
GO:0008305	integral to plasma membrane
GO:0008324	cation transmembrane transporter activity
GO:0008406	gonad development
GO:0008507	sodium:iodide symporter activity
GO:0008509	anion transmembrane transporter activity
GO:0008543	fibroblast growth factor receptor signaling pathway
GO:0008544	ectoderm development
GO:0008586	imaginal disc-derived wing morphogenesis
GO:0009253	peptidoglycan catabolic process
GO:0009309	amine biosynthetic process
GO:0009314	response to radiation
GO:0009416	response to light stimulus
GO:0009581	detection of external stimulus
GO:0009582	detection of abiotic stimulus
GO:0009583	detection of light stimulus
GO:0009584	detection of visible light
GO:0009586	rhodopsin mediated phototransduction
GO:0009595	detection of biotic stimulus
GO:0009605	response to external stimulus
GO:0009607	response to biotic stimulus
GO:0009611	response to wounding
GO:0009617	response to bacterium
GO:0009628	response to abiotic stimulus
GO:0009653	anatomical structure development
GO:0009791	multicellular organismal development
GO:0009881	photoreceptor activity
GO:0009887	organ morphogenesis
GO:0009888	organ development
GO:0009954	proximal/distal pattern formation

GO:0009991	response to extracellular stimulus
GO:0010160	formation of organ boundary
GO:0014706	striated muscle development
GO:0015020	glucuronosyltransferase activity
GO:0015075	ion transmembrane transporter activity
GO:0015101	organic cation transmembrane transporter activity
GO:0015116	sulfate transmembrane transporter activity
GO:0015145	monosaccharide transmembrane transporter activity
GO:0015179	L-amino acid transmembrane transporter activity
GO:0015291	secondary active transmembrane transporter activity
GO:0015293	symporter activity
GO:0015294	solute:cation symporter activity
GO:0015296	anion:cation symporter activity
GO:0015355	secondary active monocarboxylate transmembrane transporter activity
GO:0015370	solute:sodium symporter activity
GO:0015373	monovalent anion:sodium symporter activity
GO:0015381	high affinity sulfate transmembrane transporter activity
GO:0015629	actin cytoskeleton
GO:0015698	inorganic anion transport
GO:0016020	membrane
GO:0016021	integral to membrane
GO:0016023	cytoplasmic membrane-bound vesicle
GO:0016027	rhabdomere
GO:0016028	rhabdomere
GO:0016044	membrane organization and biogenesis
GO:0016045	detection of bacterium
GO:0016059	deactivation of rhodopsin mediated signaling
GO:0016079	neurotransmitter secretion
GO:0016284	alanine aminopeptidase activity
GO:0016459	actin cytoskeleton
GO:0016460	myosin II complex
GO:0016477	cell migration
GO:0016511	endothelin-converting enzyme activity
GO:0016566	specific transcriptional repressor activity
GO:0016787	hydrolase activity
GO:0016791	phosphoric monoester hydrolase activity
GO:0016810	hydrolase activity, acting on carbon-nitrogen (but not peptide) bonds
GO:0016811	hydrolase activity, acting on carbon-nitrogen (but not peptide) bonds, in linear amides
GO:0016814	hydrolase activity, acting on carbon-nitrogen (but not peptide) bonds, in cyclic amidines
GO:0017171	serine hydrolase activity
GO:0019226	transmission of nerve impulse
GO:0019722	calcium-mediated signaling
GO:0019730	antimicrobial humoral response
GO:0019731	antibacterial humoral response
GO:0019748	secondary metabolic process
GO:0019897	extrinsic to plasma membrane
GO:0019898	extrinsic to membrane
GO:0019932	second-messenger-mediated signaling
GO:0021700	developmental maturation
GO:0022008	neurogenesis
GO:0022610	biological adhesion
GO:0022612	gland morphogenesis
GO:0022804	active transmembrane transporter activity
GO:0022857	transmembrane transporter activity
GO:0022891	substrate-specific transmembrane transporter activity
GO:0022892	substrate-specific transporter activity
GO:0030016	myofibril
GO:0030017	sarcomere
GO:0030029	actin filament-based process
GO:0030030	cell morphogenesis
GO:0030036	actin cytoskeleton organization and biogenesis
GO:0030054	cell junction

GO:0030154	cell differentiation
GO:0030182	generation of neurons
GO:0030239	myoblast maturation
GO:0031012	extracellular matrix
GO:0031032	actomyosin structure organization and biogenesis
GO:0031175	generation of neurons
GO:0031224	intrinsic to membrane
GO:0031226	intrinsic to plasma membrane
GO:0031410	cytoplasmic vesicle
GO:0031667	response to nutrient levels
GO:0031982	vesicle
GO:0031988	membrane-bound vesicle
GO:0032501	multicellular organismal process
GO:0032502	developmental process
GO:0032989	cellular structure morphogenesis
GO:0032990	cell part morphogenesis
GO:0035107	appendage morphogenesis
GO:0035108	limb morphogenesis
GO:0035109	imaginal disc-derived limb morphogenesis
GO:0035110	leg morphogenesis
GO:0035114	imaginal disc-derived appendage morphogenesis
GO:0035150	regulation of tube size
GO:0035151	regulation of tube size, open tracheal system
GO:0035152	regulation of tube architecture, open tracheal system
GO:0035153	epithelial cell type specification, open tracheal system
GO:0035158	regulation of tube size, open tracheal system
GO:0035159	regulation of tube size, open tracheal system
GO:0035161	imaginal disc development
GO:0035215	genital disc development
GO:0035216	haltere disc development
GO:0035218	leg disc development
GO:0035220	wing disc development
GO:0035221	genital disc development
GO:0035222	wing disc pattern formation
GO:0035224	genital disc anterior/posterior pattern formation
GO:0035239	tube morphogenesis
GO:0035264	multicellular organism growth
GO:0035272	exocrine system development
GO:0035277	spiracle morphogenesis, open tracheal system
GO:0035285	appendage morphogenesis
GO:0035286	leg segmentation
GO:0035290	blastoderm segmentation
GO:0035292	blastoderm segmentation
GO:0035295	tube development
GO:0040008	regulation of growth
GO:0040014	regulation of multicellular organism growth
GO:0040018	positive regulation of multicellular organism growth
GO:0042060	wound healing
GO:0042063	gliogenesis
GO:0042133	regulation of neurotransmitter levels
GO:0042136	neurotransmitter biosynthetic process
GO:0042330	taxis
GO:0042398	amino acid derivative biosynthetic process
GO:0042445	hormone metabolic process
GO:0042578	phosphoric ester hydrolase activity
GO:0042592	homeostatic process
GO:0042598	vesicular fraction
GO:0042692	muscle cell differentiation
GO:0042706	eye photoreceptor cell fate commitment
GO:0042742	defense response to bacterium
GO:0042802	identical protein binding
GO:0042803	protein homodimerization activity

GO:0042834	peptidoglycan binding
GO:0042995	cell projection
GO:0043043	peptide biosynthetic process
GO:0043062	extracellular structure organization and biogenesis
GO:0043167	ion binding
GO:0043169	cation binding
GO:0043190	ATP-binding cassette (ABC) transporter complex
GO:0043292	contractile fiber
GO:0043297	apical junction assembly
GO:0044255	cellular lipid metabolic process
GO:0044271	nitrogen compound biosynthetic process
GO:0044420	extracellular matrix
GO:0044421	extracellular region
GO:0044425	membrane
GO:0044449	contractile fiber
GO:0044459	plasma membrane part
GO:0045087	innate immune response
GO:0045137	multicellular organismal development
GO:0045165	cell fate commitment
GO:0045211	postsynaptic membrane
GO:0045214	myoblast maturation
GO:0045216	intercellular junction assembly and maintenance
GO:0045445	skeletal muscle fiber development
GO:0046530	photoreceptor cell differentiation
GO:0046546	development of primary male sexual characteristics
GO:0046552	photoreceptor cell fate commitment
GO:0046660	multicellular organismal development
GO:0046661	multicellular organismal development
GO:0046872	metal ion binding
GO:0046943	carboxylic acid transmembrane transporter activity
GO:0046983	protein dimerization activity
GO:0048015	phosphoinositide-mediated signaling
GO:0048066	pigmentation during development
GO:0048190	wing disc dorsal/ventral pattern formation
GO:0048332	mesoderm morphogenesis
GO:0048333	mesodermal cell differentiation
GO:0048468	cell development
GO:0048469	cell development
GO:0048511	rhythmic process
GO:0048512	circadian behavior
GO:0048513	organ development
GO:0048546	digestive tract morphogenesis
GO:0048547	gut morphogenesis
GO:0048565	gut development
GO:0048567	ectodermal gut morphogenesis
GO:0048598	embryonic morphogenesis
GO:0048608	reproductive structure development
GO:0048627	skeletal muscle fiber development
GO:0048628	myoblast maturation
GO:0048645	organ formation
GO:0048646	anatomical structure development
GO:0048663	generation of neurons
GO:0048664	generation of neurons
GO:0048666	generation of neurons
GO:0048667	neuron morphogenesis during differentiation
GO:0048699	generation of neurons
GO:0048729	tissue morphogenesis
GO:0048731	system development
GO:0048732	gland development
GO:0048736	appendage development
GO:0048737	imaginal disc-derived appendage development
GO:0048741	skeletal muscle fiber development

GO:0048747	muscle fiber development
GO:0048806	genitalia development
GO:0048812	neuron morphogenesis during differentiation
GO:0048856	anatomical structure development
GO:0048858	cell morphogenesis
GO:0048859	formation of anatomical boundary
GO:0048869	cellular developmental process
GO:0050768	negative regulation of neurogenesis
GO:0050789	regulation of biological process
GO:0050790	regulation of catalytic activity
GO:0050793	regulation of developmental process
GO:0050808	synapse organization and biogenesis
GO:0050830	defense response to Gram-positive bacterium
GO:0050832	defense response to fungus
GO:0050877	neurological system process
GO:0050878	regulation of body fluid levels
GO:0050896	response to stimulus
GO:0050906	detection of stimulus during sensory perception
GO:0050908	detection of light stimulus during visual perception
GO:0050953	sensory perception of light stimulus
GO:0050962	detection of light stimulus during sensory perception
GO:0051093	negative regulation of developmental process
GO:0051146	striated muscle cell differentiation
GO:0051179	localization
GO:0051234	establishment of localization
GO:0051606	detection of stimulus
GO:0051674	localization of cell
GO:0051704	multi-organism process
GO:0051707	response to other organism
GO:0055001	muscle cell development
GO:0055002	striated muscle cell development
GO:0055074	calcium ion homeostasis
GO:0060173	limb development
GO:0065007	biological regulation
GO:0065008	regulation of biological quality
GO:0065009	regulation of a molecular function

Table S1B: Gene Ontologies Down-regulated in yw, w1118 DR. All gene ontologies have at least 5 genes in the GO and p value < 0.05.

GO:0000070	M phase of mitotic cell cycle
GO:0000074	regulation of progression through cell cycle
GO:0000075	cell cycle checkpoint
GO:0000077	DNA damage checkpoint
GO:0000082	interphase of mitotic cell cycle
GO:0000084	interphase of mitotic cell cycle
GO:0000087	M phase of mitotic cell cycle
GO:0000119	DNA-directed RNA polymerase II, holoenzyme
GO:0000122	negative regulation of transcription from RNA polymerase II promoter
GO:0000226	microtubule cytoskeleton organization and biogenesis
GO:0000228	nuclear chromosome
GO:0000278	mitotic cell cycle
GO:0000279	M phase
GO:0000375	RNA splicing, via transesterification reactions
GO:0000377	RNA splicing, via transesterification reactions with bulged adenosine as nucleophile
GO:0000380	alternative nuclear mRNA splicing, via spliceosome
GO:0000381	regulation of alternative nuclear mRNA splicing, via spliceosome
GO:0000398	nuclear mRNA splicing, via spliceosome
GO:0000578	embryonic axis specification
GO:0000775	chromosome, pericentric region
GO:0000776	chromosome, pericentric region

GO:0000777	condensed chromosome, pericentric region
GO:0000779	condensed chromosome, pericentric region
GO:0000785	chromatin
GO:0000792	heterochromatin
GO:0000793	condensed chromosome
GO:0000796	condensin complex
GO:0000808	origin recognition complex
GO:0000819	sister chromatid segregation
GO:0000910	cytokinesis
GO:0000940	condensed chromosome, pericentric region
GO:0003002	regionalization
GO:0003676	nucleic acid binding
GO:0003677	DNA binding
GO:0003678	DNA helicase activity
GO:0003682	chromatin binding
GO:0003702	RNA polymerase II transcription factor activity
GO:0003712	transcription cofactor activity
GO:0003713	transcription coactivator activity
GO:0003723	RNA binding
GO:0003729	mRNA binding
GO:0003777	microtubule motor activity
GO:0003887	DNA-directed DNA polymerase activity
GO:0004003	ATP-dependent DNA helicase activity
GO:0004004	ATP-dependent RNA helicase activity
GO:0004386	helicase activity
GO:0004402	histone acetyltransferase activity
GO:0004468	lysine N-acetyltransferase activity
GO:0004518	nuclease activity
GO:0004519	endonuclease activity
GO:0004520	endodeoxyribonuclease activity
GO:0004521	endoribonuclease activity
GO:0004529	exodeoxyribonuclease activity
GO:0004536	deoxyribonuclease activity
GO:0004672	protein kinase activity
GO:0004674	protein serine/threonine kinase activity
GO:0004693	cyclin-dependent protein kinase activity
GO:0004721	phosphoprotein phosphatase activity
GO:0005515	protein binding
GO:0005622	intracellular
GO:0005623	cell
GO:0005634	nucleus
GO:0005635	nuclear envelope
GO:0005654	nuclear lumen
GO:0005656	pre-replicative complex
GO:0005657	replication fork
GO:0005658	alpha DNA polymerase:primase complex
GO:0005663	DNA replication factor C complex
GO:0005664	nuclear origin of replication recognition complex
GO:0005667	transcription factor complex
GO:0005669	DNA-directed RNA polymerase II, holoenzyme
GO:0005678	chromatin assembly complex
GO:0005694	chromosome
GO:0005700	polytene chromosome
GO:0005813	centrosome
GO:0005815	microtubule organizing center
GO:0005819	spindle
GO:0005828	kinetochore microtubule
GO:0005856	cytoskeleton
GO:0005871	kinesin complex
GO:0005874	microtubule
GO:0005876	spindle microtubule
GO:0006139	nucleobase, nucleoside, nucleotide and nucleic acid metabolic process

GO:0006259	DNA metabolic process
GO:0006260	DNA replication
GO:0006261	DNA-dependent DNA replication
GO:0006267	pre-replicative complex assembly
GO:0006270	DNA-dependent DNA replication
GO:0006271	DNA-dependent DNA replication
GO:0006275	regulation of DNA replication
GO:0006277	DNA amplification
GO:0006281	DNA repair
GO:0006289	nucleotide-excision repair
GO:0006310	DNA recombination
GO:0006334	nucleosome assembly
GO:0006350	transcription
GO:0006351	transcription, DNA-dependent
GO:0006352	transcription initiation
GO:0006355	regulation of transcription, DNA-dependent
GO:0006357	regulation of transcription from RNA polymerase II promoter
GO:0006359	regulation of transcription from RNA polymerase III promoter
GO:0006366	transcription from RNA polymerase II promoter
GO:0006367	transcription initiation from RNA polymerase II promoter
GO:0006396	RNA processing
GO:0006397	mRNA processing
GO:0006464	protein modification process
GO:0006468	protein amino acid phosphorylation
GO:0006470	protein amino acid dephosphorylation
GO:0006606	protein import into nucleus
GO:0006793	phosphorus metabolic process
GO:0006796	phosphate metabolic process
GO:0006950	response to stress
GO:0006974	response to DNA damage stimulus
GO:0006996	organelle organization and biogenesis
GO:0007010	cytoskeleton organization and biogenesis
GO:0007017	microtubule-based process
GO:0007049	cell cycle
GO:0007051	spindle organization and biogenesis
GO:0007052	mitotic spindle organization and biogenesis
GO:0007059	chromosome segregation
GO:0007067	M phase of mitotic cell cycle
GO:0007076	M phase of mitotic cell cycle
GO:0007088	regulation of mitosis
GO:0007093	mitotic cell cycle checkpoint
GO:0007094	mitotic cell cycle spindle assembly checkpoint
GO:0007098	centrosome cycle
GO:0007100	mitotic centrosome separation
GO:0007126	M phase of meiotic cell cycle
GO:0007127	M phase of meiotic cell cycle
GO:0007131	M phase of meiotic cell cycle
GO:0007143	female meiosis
GO:0007275	multicellular organismal development
GO:0007276	gamete generation
GO:0007280	pole cell migration
GO:0007281	germ cell development
GO:0007292	female gamete generation
GO:0007293	germarium-derived egg chamber formation
GO:0007294	germarium-derived oocyte fate determination
GO:0007304	ovarian follicle cell development
GO:0007306	ovarian follicle cell development
GO:0007307	ovarian follicle cell development
GO:0007311	maternal determination of dorsal/ventral axis, oocyte, germ-line encoded
GO:0007346	regulation of progression through mitotic cell cycle
GO:0007348	regulation of progression through embryonic mitotic cell cycle
GO:0007349	cellularization

GO:0007351	blastoderm segmentation
GO:0007389	multicellular organismal development
GO:0007568	aging
GO:0008026	ATP-dependent helicase activity
GO:0008063	Toll signaling pathway
GO:0008094	DNA-dependent ATPase activity
GO:0008134	transcription factor binding
GO:0008156	negative regulation of DNA replication
GO:0008168	methyltransferase activity
GO:0008186	RNA-dependent ATPase activity
GO:0008276	protein methyltransferase activity
GO:0008283	cell proliferation
GO:0008296	3'-5'-exodeoxyribonuclease activity
GO:0008340	multicellular organismal aging
GO:0008354	germ cell migration
GO:0008356	asymmetric cell division
GO:0008375	acetylglucosaminyltransferase activity
GO:0008380	RNA splicing
GO:0008574	plus-end-directed microtubule motor activity
GO:0008595	blastoderm segmentation
GO:0009653	anatomical structure development
GO:0009719	response to endogenous stimulus
GO:0009790	embryonic development
GO:0009794	regulation of progression through embryonic mitotic cell cycle
GO:0009798	axis specification
GO:0009880	embryonic development
GO:0009892	negative regulation of metabolic process
GO:0009893	positive regulation of metabolic process
GO:0009948	anterior/posterior axis specification
GO:0009949	anterior/posterior axis specification
GO:0009950	dorsal/ventral axis specification
GO:0009951	dorsal/ventral axis specification
GO:0009952	anterior/posterior pattern formation
GO:0009953	dorsal/ventral pattern formation
GO:0009994	oocyte differentiation
GO:0010259	multicellular organismal aging
GO:0010467	gene expression
GO:0010468	regulation of gene expression
GO:0015630	microtubule cytoskeleton
GO:0016015	morphogen activity
GO:0016043	cellular component organization and biogenesis
GO:0016070	RNA metabolic process
GO:0016071	mRNA metabolic process
GO:0016251	general RNA polymerase II transcription factor activity
GO:0016301	kinase activity
GO:0016310	phosphorylation
GO:0016311	dephosphorylation
GO:0016321	female meiosis
GO:0016455	RNA polymerase II transcription mediator activity
GO:0016481	negative regulation of transcription
GO:0016538	cyclin-dependent protein kinase regulator activity
GO:0016563	transcription activator activity
GO:0016564	transcription repressor activity
GO:0016568	chromatin modification
GO:0016569	covalent chromatin modification
GO:0016570	histone modification
GO:0016572	histone phosphorylation
GO:0016591	DNA-directed RNA polymerase II, holoenzyme
GO:0016740	transferase activity
GO:0016772	transferase activity, transferring phosphorus-containing groups
GO:0016773	phosphotransferase activity, alcohol group as acceptor
GO:0016779	nucleotidyltransferase activity

GO:0016788	hydrolase activity, acting on ester bonds
GO:0016791	phosphoric monoester hydrolase activity
GO:0016799	hydrolase activity, hydrolyzing N-glycosyl compounds
GO:0016895	exodeoxyribonuclease activity, producing 5'-phosphomonoesters
GO:0017038	protein import
GO:0019104	DNA N-glycosylase activity
GO:0019219	regulation of nucleobase, nucleoside, nucleotide and nucleic acid metabolic process
GO:0019220	regulation of phosphate metabolic process
GO:0019222	regulation of metabolic process
GO:0019953	sexual reproduction
GO:0022402	cell cycle
GO:0022403	cell cycle phase
GO:0022607	cellular component assembly
GO:0022616	DNA strand elongation
GO:0030154	cell differentiation
GO:0030261	chromosome condensation
GO:0030496	midbody
GO:0030703	eggshell formation
GO:0030706	germarium-derived oocyte differentiation
GO:0030707	ovarian follicle cell development
GO:0030716	oocyte fate determination
GO:0030717	karyosome formation
GO:0030720	germarium-derived egg chamber formation
GO:0030727	germarium-derived female germ-line cyst formation
GO:0030894	replication fork
GO:0031023	microtubule organizing center organization and biogenesis
GO:0031323	regulation of cellular metabolic process
GO:0031324	negative regulation of cellular metabolic process
GO:0031325	positive regulation of cellular metabolic process
GO:0031519	PcG protein complex
GO:0031523	Myb complex
GO:0031570	DNA integrity checkpoint
GO:0031577	spindle checkpoint
GO:0031965	nuclear envelope
GO:0031974	membrane-enclosed lumen
GO:0031981	nuclear lumen
GO:0032502	developmental process
GO:0032774	RNA biosynthetic process
GO:0032837	distributive segregation
GO:0032991	macromolecular complex
GO:0033036	macromolecule localization
GO:0033301	cell cycle comprising mitosis without cytokinesis
GO:0035058	sensory cilium biogenesis
GO:0035098	ESC/E(Z) complex
GO:0035102	PRC1 complex
GO:0035186	syncytial blastoderm mitotic cell cycle
GO:0035303	regulation of dephosphorylation
GO:0035304	regulation of protein amino acid dephosphorylation
GO:0042023	DNA endoreduplication
GO:0042054	histone methyltransferase activity
GO:0042384	cilium biogenesis
GO:0042575	DNA polymerase complex
GO:0042578	phosphoric ester hydrolase activity
GO:0042770	DNA damage response, signal transduction
GO:0043138	3'-5' DNA helicase activity
GO:0043170	macromolecule metabolic process
GO:0043189	H4/H2A histone acetyltransferase complex
GO:0043226	organelle
GO:0043227	membrane-bound organelle
GO:0043228	non-membrane-bound organelle
GO:0043229	intracellular organelle
GO:0043231	intracellular membrane-bound organelle

GO:0043232	intracellular non-membrane-bound organelle
GO:0043233	organelle lumen
GO:0043234	protein complex
GO:0043283	biopolymer metabolic process
GO:0043412	biopolymer modification
GO:0043414	biopolymer methylation
GO:0043596	nuclear replication fork
GO:0043601	nuclear replication fork
GO:0043623	cellular protein complex assembly
GO:0043687	post-translational protein modification
GO:0044237	cellular metabolic process
GO:0044238	primary metabolic process
GO:0044422	organelle
GO:0044424	intracellular
GO:0044427	chromosome
GO:0044428	nucleus
GO:0044430	cytoskeleton
GO:0044446	intracellular organelle
GO:0044450	microtubule organizing center
GO:0044451	nuclear lumen
GO:0044453	nuclear envelope
GO:0044454	nuclear chromosome
GO:0044464	cell
GO:0045132	M phase of meiotic cell cycle
GO:0045143	M phase of meiotic cell cycle
GO:0045448	mitotic cell cycle, embryonic
GO:0045449	regulation of transcription
GO:0045786	negative regulation of progression through cell cycle
GO:0045787	positive regulation of progression through cell cycle
GO:0045892	negative regulation of transcription, DNA-dependent
GO:0045893	positive regulation of transcription, DNA-dependent
GO:0045931	positive regulation of progression through mitotic cell cycle
GO:0045934	negative regulation of nucleobase, nucleoside, nucleotide and nucleic acid metabolic process
GO:0045935	positive regulation of nucleobase, nucleoside, nucleotide and nucleic acid metabolic process
GO:0045941	positive regulation of transcription
GO:0046823	negative regulation of nucleocytoplasmic transport
GO:0046974	histone lysine N-methyltransferase activity (H3-K9 specific)
GO:0046976	histone lysine N-methyltransferase activity (H3-K27 specific)
GO:0048024	regulation of nuclear mRNA splicing, via spliceosome
GO:0048134	germ cell development
GO:0048135	female germ-line cyst formation
GO:0048468	cell development
GO:0048477	oogenesis
GO:0048518	positive regulation of biological process
GO:0048519	negative regulation of biological process
GO:0048522	positive regulation of cellular process
GO:0048523	negative regulation of cellular process
GO:0048646	anatomical structure development
GO:0048856	anatomical structure development
GO:0048869	cellular developmental process
GO:0050000	chromosome localization
GO:0050684	regulation of mRNA processing
GO:0050789	regulation of biological process
GO:0050794	regulation of cellular process
GO:0051051	negative regulation of transport
GO:0051052	regulation of DNA metabolic process
GO:0051053	negative regulation of DNA metabolic process
GO:0051170	nuclear import
GO:0051174	regulation of phosphorus metabolic process
GO:0051224	negative regulation of protein transport
GO:0051225	spindle assembly
GO:0051246	regulation of protein metabolic process

GO:0051248	negative regulation of protein metabolic process
GO:0051252	regulation of RNA metabolic process
GO:0051276	chromosome organization and biogenesis
GO:0051297	centrosome organization and biogenesis
GO:0051299	centrosome cycle
GO:0051301	cell division
GO:0051303	establishment of chromosome localization
GO:0051320	interphase
GO:0051321	meiotic cell cycle
GO:0051322	anaphase
GO:0051325	interphase
GO:0051327	M phase of meiotic cell cycle
GO:0051329	interphase of mitotic cell cycle
GO:0051726	regulation of cell cycle
GO:0065001	specification of axis polarity
GO:0065003	macromolecular complex assembly
GO:0065004	protein-DNA complex assembly
GO:0065007	biological regulation

Table S1C: Gene Ontologies Up-regulated in Canton-S DR. All gene ontologies have at least 5 genes in the GO and p value < 0.05.

GO:0000038	very-long-chain fatty acid metabolic process
GO:0000267	cell fraction
GO:0000902	cell morphogenesis
GO:0000904	cell development
GO:0001505	regulation of neurotransmitter levels
GO:0001736	establishment of planar polarity
GO:0001738	morphogenesis of a polarized epithelium
GO:0001751	compound eye photoreceptor cell differentiation
GO:0001752	compound eye photoreceptor fate commitment
GO:0001754	eye photoreceptor cell differentiation
GO:0001763	morphogenesis of a branching structure
GO:0002165	instar larval or pupal development
GO:0002376	immune system process
GO:0003008	system process
GO:0003779	actin binding
GO:0003993	acid phosphatase activity
GO:0004035	alkaline phosphatase activity
GO:0004046	aminoacylase activity
GO:0004175	endopeptidase activity
GO:0004197	cysteine-type endopeptidase activity
GO:0004263	chymotrypsin activity
GO:0004312	fatty-acid synthase activity
GO:0004568	chitinase activity
GO:0004672	protein kinase activity
GO:0004674	protein serine/threonine kinase activity
GO:0004723	calcium-dependent protein serine/threonine phosphatase activity
GO:0004857	enzyme inhibitor activity
GO:0004871	signal transducer activity
GO:0004872	receptor activity
GO:0004888	transmembrane receptor activity
GO:0005102	receptor binding
GO:0005160	transforming growth factor beta receptor binding
GO:0005184	neuropeptide hormone activity
GO:0005200	structural constituent of cytoskeleton
GO:0005275	amine transmembrane transporter activity
GO:0005283	sodium:amino acid symporter activity
GO:0005316	high affinity inorganic phosphate:sodium symporter activity
GO:0005328	neurotransmitter:sodium symporter activity
GO:0005342	organic acid transmembrane transporter activity

GO:0005388	calcium-transporting ATPase activity
GO:0005416	cation:amino acid symporter activity
GO:0005436	sodium:phosphate symporter activity
GO:0005509	calcium ion binding
GO:0005515	protein binding
GO:0005516	calmodulin binding
GO:0005578	proteinaceous extracellular matrix
GO:0005604	basement membrane
GO:0005615	extracellular space
GO:0005624	membrane fraction
GO:0005792	microsome
GO:0005856	cytoskeleton
GO:0005859	muscle myosin complex
GO:0005865	sarcomere
GO:0005886	plasma membrane
GO:0005887	integral to plasma membrane
GO:0006468	protein amino acid phosphorylation
GO:0006536	glutamate metabolic process
GO:0006568	tryptophan metabolic process
GO:0006633	fatty acid biosynthetic process
GO:0006793	phosphorus metabolic process
GO:0006796	phosphate metabolic process
GO:0006810	transport
GO:0006811	ion transport
GO:0006812	cation transport
GO:0006816	calcium ion transport
GO:0006817	phosphate transport
GO:0006820	anion transport
GO:0006874	cellular calcium ion homeostasis
GO:0006928	cell motility
GO:0006952	defense response
GO:0006955	immune response
GO:0007154	cell communication
GO:0007155	cell adhesion
GO:0007164	establishment of tissue polarity
GO:0007165	signal transduction
GO:0007166	cell surface receptor linked signal transduction
GO:0007167	enzyme linked receptor protein signaling pathway
GO:0007169	transmembrane receptor protein tyrosine kinase signaling pathway
GO:0007186	G-protein coupled receptor protein signaling pathway
GO:0007200	G-protein signaling, coupled to IP3 second messenger (phospholipase C activating)
GO:0007218	neuropeptide signaling pathway
GO:0007242	intracellular signaling cascade
GO:0007267	cell-cell signaling
GO:0007268	synaptic transmission
GO:0007275	multicellular organismal development
GO:0007392	dorsal closure
GO:0007399	nervous system development
GO:0007409	axonogenesis
GO:0007411	axonogenesis
GO:0007416	synaptogenesis
GO:0007424	open tracheal system development
GO:0007431	salivary gland development
GO:0007432	salivary gland boundary specification
GO:0007439	ectodermal gut development
GO:0007447	imaginal disc development
GO:0007458	eye-antennal disc morphogenesis
GO:0007472	wing disc morphogenesis
GO:0007473	wing disc proximal/distal pattern formation
GO:0007476	imaginal disc-derived wing morphogenesis
GO:0007478	leg disc morphogenesis
GO:0007498	mesoderm development

GO:0007507	heart development
GO:0007517	muscle development
GO:0007519	skeletal muscle development
GO:0007525	somatic muscle development
GO:0007528	skeletal muscle fiber development
GO:0007548	sex differentiation
GO:0007559	histolysis
GO:0007601	visual perception
GO:0007602	phototransduction
GO:0007604	phototransduction, UV
GO:0007623	circadian rhythm
GO:0007626	locomotory behavior
GO:0008020	G-protein coupled photoreceptor activity
GO:0008021	synaptic vesicle
GO:0008083	growth factor activity
GO:0008084	imaginal disc growth factor activity
GO:0008092	cytoskeletal protein binding
GO:0008253	5'-nucleotidase activity
GO:0008271	secondary active sulfate transmembrane transporter activity
GO:0008277	regulation of G-protein coupled receptor protein signaling pathway
GO:0008289	lipid binding
GO:0008324	cation transmembrane transporter activity
GO:0008360	regulation of cell shape
GO:0008367	bacterial binding
GO:0008368	Gram-negative bacterial binding
GO:0008507	sodium:iodide symporter activity
GO:0008509	anion transmembrane transporter activity
GO:0008514	organic anion transmembrane transporter activity
GO:0008523	sodium-dependent multivitamin transmembrane transporter activity
GO:0008543	fibroblast growth factor receptor signaling pathway
GO:0008586	imaginal disc-derived wing morphogenesis
GO:0008610	lipid biosynthetic process
GO:0009055	electron carrier activity
GO:0009064	glutamine family amino acid metabolic process
GO:0009065	glutamine family amino acid catabolic process
GO:0009309	amine biosynthetic process
GO:0009314	response to radiation
GO:0009411	response to UV
GO:0009416	response to light stimulus
GO:0009581	detection of external stimulus
GO:0009582	detection of abiotic stimulus
GO:0009583	detection of light stimulus
GO:0009584	detection of visible light
GO:0009586	rhodopsin mediated phototransduction
GO:0009589	detection of UV
GO:0009595	detection of biotic stimulus
GO:0009605	response to external stimulus
GO:0009607	response to biotic stimulus
GO:0009617	response to bacterium
GO:0009628	response to abiotic stimulus
GO:0009791	multicellular organismal development
GO:0009881	photoreceptor activity
GO:0009888	organ development
GO:0014706	striated muscle development
GO:0015075	ion transmembrane transporter activity
GO:0015085	calcium ion transmembrane transporter activity
GO:0015101	organic cation transmembrane transporter activity
GO:0015103	inorganic anion transmembrane transporter activity
GO:0015114	phosphate transmembrane transporter activity
GO:0015116	sulfate transmembrane transporter activity
GO:0015171	amino acid transmembrane transporter activity
GO:0015179	L-amino acid transmembrane transporter activity

GO:0015291	secondary active transmembrane transporter activity
GO:0015293	symporter activity
GO:0015294	solute:cation symporter activity
GO:0015296	anion:cation symporter activity
GO:0015370	solute:sodium symporter activity
GO:0015373	monovalent anion:sodium symporter activity
GO:0015381	high affinity sulfate transmembrane transporter activity
GO:0015399	primary active transmembrane transporter activity
GO:0015405	P-P-bond-hydrolysis-driven transmembrane transporter activity
GO:0015629	actin cytoskeleton
GO:0015674	di-, tri-valent inorganic cation transport
GO:0015698	inorganic anion transport
GO:0015711	organic anion transport
GO:0016020	membrane
GO:0016021	integral to membrane
GO:0016023	cytoplasmic membrane-bound vesicle
GO:0016027	rhabdomere
GO:0016028	rhabdomere
GO:0016045	detection of bacterium
GO:0016053	organic acid biosynthetic process
GO:0016056	rhodopsin mediated signaling
GO:0016059	deactivation of rhodopsin mediated signaling
GO:0016271	tissue death
GO:0016301	kinase activity
GO:0016310	phosphorylation
GO:0016459	actin cytoskeleton
GO:0016460	myosin II complex
GO:0016477	cell migration
GO:0016511	endothelin-converting enzyme activity
GO:0016772	transferase activity, transferring phosphorus-containing groups
GO:0016773	phosphotransferase activity, alcohol group as acceptor
GO:0016787	hydrolase activity
GO:0016788	hydrolase activity, acting on ester bonds
GO:0016791	phosphoric monoester hydrolase activity
GO:0016810	hydrolase activity, acting on carbon-nitrogen (but not peptide) bonds
GO:0016811	hydrolase activity, acting on carbon-nitrogen (but not peptide) bonds, in linear amides
GO:0016820	hydrolase activity, acting on acid anhydrides, catalyzing transmembrane movement of substances
GO:0016830	carbon-carbon lyase activity
GO:0016831	carboxy-lyase activity
GO:0019201	nucleotide kinase activity
GO:0019226	transmission of nerve impulse
GO:0019722	calcium-mediated signaling
GO:0019725	cellular homeostasis
GO:0019897	extrinsic to plasma membrane
GO:0019898	extrinsic to membrane
GO:0019932	second-messenger-mediated signaling
GO:0019992	diacylglycerol binding
GO:0021700	developmental maturation
GO:0022008	neurogenesis
GO:0022410	circadian sleep/wake cycle
GO:0022603	regulation of anatomical structure morphogenesis
GO:0022604	regulation of cell morphogenesis
GO:0022610	biological adhesion
GO:0022804	active transmembrane transporter activity
GO:0022857	transmembrane transporter activity
GO:0022891	substrate-specific transmembrane transporter activity
GO:0022892	substrate-specific transporter activity
GO:0030001	metal ion transport
GO:0030016	myofibril
GO:0030017	sarcomere
GO:0030029	actin filament-based process
GO:0030030	cell morphogenesis

GO:0030036	actin cytoskeleton organization and biogenesis
GO:0030136	clathrin-coated vesicle
GO:0030154	cell differentiation
GO:0030182	generation of neurons
GO:0030239	myoblast maturation
GO:0030414	protease inhibitor activity
GO:0031012	extracellular matrix
GO:0031175	generation of neurons
GO:0031224	intrinsic to membrane
GO:0031226	intrinsic to plasma membrane
GO:0031410	cytoplasmic vesicle
GO:0031982	vesicle
GO:0031988	membrane-bound vesicle
GO:0032501	multicellular organismal process
GO:0032989	cellular structure morphogenesis
GO:0032990	cell part morphogenesis
GO:0035026	leading edge cell differentiation
GO:0035070	salivary gland development
GO:0035071	salivary gland cell autophagic cell death
GO:0035107	appendage morphogenesis
GO:0035114	imaginal disc-derived appendage morphogenesis
GO:0035146	branching morphogenesis of a tube
GO:0035147	branch fusion, open tracheal system
GO:0035152	regulation of tube architecture, open tracheal system
GO:0035153	epithelial cell type specification, open tracheal system
GO:0035154	terminal cell fate specification, open tracheal system
GO:0035215	genital disc development
GO:0035216	haltere disc development
GO:0035218	leg disc development
GO:0035220	wing disc development
GO:0035222	wing disc pattern formation
GO:0035272	exocrine system development
GO:0035277	spiracle morphogenesis, open tracheal system
GO:0042044	fluid transport
GO:0042133	regulation of neurotransmitter levels
GO:0042136	neurotransmitter biosynthetic process
GO:0042330	taxis
GO:0042398	amino acid derivative biosynthetic process
GO:0042445	hormone metabolic process
GO:0042446	hormone biosynthetic process
GO:0042461	photoreceptor cell development
GO:0042578	phosphoric ester hydrolase activity
GO:0042592	homeostatic process
GO:0042598	vesicular fraction
GO:0042626	ATPase activity, coupled to transmembrane movement of substances
GO:0042692	muscle cell differentiation
GO:0042706	eye photoreceptor cell fate commitment
GO:0042742	defense response to bacterium
GO:0042745	circadian sleep/wake cycle
GO:0042749	regulation of circadian sleep/wake cycle
GO:0042752	regulation of circadian rhythm
GO:0042995	cell projection
GO:0043167	ion binding
GO:0043169	cation binding
GO:0043190	ATP-binding cassette (ABC) transporter complex
GO:0043292	contractile fiber
GO:0043492	ATPase activity, coupled to movement of substances
GO:0043687	post-translational protein modification
GO:0044271	nitrogen compound biosynthetic process
GO:0044420	extracellular matrix
GO:0044421	extracellular region
GO:0044425	membrane

GO:0044449	contractile fiber
GO:0044456	synapse
GO:0044459	plasma membrane part
GO:0045137	multicellular organismal development
GO:0045165	cell fate commitment
GO:0045187	regulation of circadian sleep/wake cycle, sleep
GO:0045211	postsynaptic membrane
GO:0045445	skeletal muscle fiber development
GO:0045893	positive regulation of transcription, DNA-dependent
GO:0045935	positive regulation of nucleobase, nucleoside, nucleotide and nucleic acid metabolic process
GO:0045941	positive regulation of transcription
GO:0046394	carboxylic acid biosynthetic process
GO:0046530	photoreceptor cell differentiation
GO:0046552	photoreceptor cell fate commitment
GO:0046661	multicellular organismal development
GO:0046663	dorsal closure, leading edge cell differentiation
GO:0046872	metal ion binding
GO:0046943	carboxylic acid transmembrane transporter activity
GO:0046983	protein dimerization activity
GO:0048015	phosphoinositide-mediated signaling
GO:0048102	autophagic cell death
GO:0048468	cell development
GO:0048469	cell development
GO:0048511	rhythmic process
GO:0048512	circadian behavior
GO:0048513	organ development
GO:0048565	gut development
GO:0048567	ectodermal gut morphogenesis
GO:0048627	skeletal muscle fiber development
GO:0048628	myoblast maturation
GO:0048666	generation of neurons
GO:0048667	neuron morphogenesis during differentiation
GO:0048699	generation of neurons
GO:0048731	system development
GO:0048732	gland development
GO:0048736	appendage development
GO:0048737	imaginal disc-derived appendage development
GO:0048741	skeletal muscle fiber development
GO:0048747	muscle fiber development
GO:0048754	branching morphogenesis of a tube
GO:0048812	neuron morphogenesis during differentiation
GO:0048856	anatomical structure development
GO:0048858	cell morphogenesis
GO:0048869	cellular developmental process
GO:0050789	regulation of biological process
GO:0050790	regulation of catalytic activity
GO:0050793	regulation of developmental process
GO:0050802	circadian sleep/wake cycle, sleep
GO:0050808	synapse organization and biogenesis
GO:0050877	neurological system process
GO:0050896	response to stimulus
GO:0050906	detection of stimulus during sensory perception
GO:0050908	detection of light stimulus during visual perception
GO:0050953	sensory perception of light stimulus
GO:0050962	detection of light stimulus during sensory perception
GO:0051146	striated muscle cell differentiation
GO:0051179	localization
GO:0051180	vitamin transport
GO:0051234	establishment of localization
GO:0051235	localization
GO:0051336	regulation of hydrolase activity
GO:0051606	detection of stimulus

GO:0051674	localization of cell
GO:0051704	multi-organism process
GO:0051707	response to other organism
GO:0055001	muscle cell development
GO:0055002	striated muscle cell development
GO:0055074	calcium ion homeostasis
GO:0060089	molecular transducer activity
GO:0065007	biological regulation
GO:0065008	regulation of biological quality
GO:0065009	regulation of a molecular function

Table S1D: Gene Ontologies Down-regulated in Canton-S DR. All gene ontologies have at least 5 genes in the GO and p value < 0.05.

GO:0000070	M phase of mitotic cell cycle
GO:0000074	regulation of progression through cell cycle
GO:0000075	cell cycle checkpoint
GO:0000079	regulation of cyclin-dependent protein kinase activity
GO:0000082	interphase of mitotic cell cycle
GO:0000084	interphase of mitotic cell cycle
GO:0000087	M phase of mitotic cell cycle
GO:0000119	DNA-directed RNA polymerase II, holoenzyme
GO:0000122	negative regulation of transcription from RNA polymerase II promoter
GO:0000152	nuclear ubiquitin ligase complex
GO:0000226	microtubule cytoskeleton organization and biogenesis
GO:0000228	nuclear chromosome
GO:0000278	mitotic cell cycle
GO:0000279	M phase
GO:0000280	nuclear division
GO:0000375	RNA splicing, via transesterification reactions
GO:0000377	RNA splicing, via transesterification reactions with bulged adenosine as nucleophile
GO:0000380	alternative nuclear mRNA splicing, via spliceosome
GO:0000381	regulation of alternative nuclear mRNA splicing, via spliceosome
GO:0000398	nuclear mRNA splicing, via spliceosome
GO:0000775	chromosome, pericentric region
GO:0000776	chromosome, pericentric region
GO:0000777	condensed chromosome, pericentric region
GO:0000779	condensed chromosome, pericentric region
GO:0000785	chromatin
GO:0000792	heterochromatin
GO:0000793	condensed chromosome
GO:0000808	origin recognition complex
GO:0000819	sister chromatid segregation
GO:0000902	cell morphogenesis
GO:0000904	cell development
GO:0000910	cytokinesis
GO:0000930	gamma-tubulin complex
GO:0000940	condensed chromosome, pericentric region
GO:0001709	cell fate commitment
GO:0002164	larval development
GO:0002165	instar larval or pupal development
GO:0002520	immune system development
GO:0003002	regionalization
GO:0003676	nucleic acid binding
GO:0003677	DNA binding
GO:0003678	DNA helicase activity
GO:0003682	chromatin binding
GO:0003702	RNA polymerase II transcription factor activity
GO:0003712	transcription cofactor activity
GO:0003713	transcription coactivator activity
GO:0003723	RNA binding

GO:0003729	mRNA binding
GO:0003730	mRNA 3'-UTR binding
GO:0003777	microtubule motor activity
GO:0003887	DNA-directed DNA polymerase activity
GO:0004003	ATP-dependent DNA helicase activity
GO:0004386	helicase activity
GO:0004518	nuclease activity
GO:0004519	endonuclease activity
GO:0004520	endodeoxyribonuclease activity
GO:0004527	exonuclease activity
GO:0004529	exodeoxyribonuclease activity
GO:0004536	deoxyribonuclease activity
GO:0004672	protein kinase activity
GO:0004674	protein serine/threonine kinase activity
GO:0004702	receptor signaling protein serine/threonine kinase activity
GO:0004708	MAP kinase kinase activity
GO:0004712	protein serine/threonine/tyrosine kinase activity
GO:0004725	protein tyrosine phosphatase activity
GO:0004843	ubiquitin-specific protease activity
GO:0005515	protein binding
GO:0005622	intracellular
GO:0005623	cell
GO:0005634	nucleus
GO:0005635	nuclear envelope
GO:0005654	nuclear lumen
GO:0005656	pre-replicative complex
GO:0005657	replication fork
GO:0005663	DNA replication factor C complex
GO:0005664	nuclear origin of replication recognition complex
GO:0005667	transcription factor complex
GO:0005669	DNA-directed RNA polymerase II, holoenzyme
GO:0005678	chromatin assembly complex
GO:0005680	anaphase-promoting complex
GO:0005694	chromosome
GO:0005700	polytene chromosome
GO:0005813	centrosome
GO:0005815	microtubule organizing center
GO:0005819	spindle
GO:0005828	kinetochore microtubule
GO:0005856	cytoskeleton
GO:0005871	kinesin complex
GO:0005874	microtubule
GO:0005876	spindle microtubule
GO:0006139	nucleobase, nucleoside, nucleotide and nucleic acid metabolic process
GO:0006259	DNA metabolic process
GO:0006260	DNA replication
GO:0006261	DNA-dependent DNA replication
GO:0006267	pre-replicative complex assembly
GO:0006270	DNA-dependent DNA replication
GO:0006275	regulation of DNA replication
GO:0006277	DNA amplification
GO:0006281	DNA repair
GO:0006289	nucleotide-excision repair
GO:0006310	DNA recombination
GO:0006323	DNA packaging
GO:0006325	establishment and/or maintenance of chromatin architecture
GO:0006334	nucleosome assembly
GO:0006338	chromatin remodeling
GO:0006350	transcription
GO:0006351	transcription, DNA-dependent
GO:0006352	transcription initiation
GO:0006355	regulation of transcription, DNA-dependent

GO:0006357	regulation of transcription from RNA polymerase II promoter
GO:0006366	transcription from RNA polymerase II promoter
GO:0006367	transcription initiation from RNA polymerase II promoter
GO:0006396	RNA processing
GO:0006397	mRNA processing
GO:0006401	RNA catabolic process
GO:0006402	mRNA catabolic process
GO:0006403	RNA localization
GO:0006405	RNA export from nucleus
GO:0006406	mRNA export from nucleus
GO:0006417	regulation of translation
GO:0006461	protein complex assembly
GO:0006464	protein modification process
GO:0006468	protein amino acid phosphorylation
GO:0006470	protein amino acid dephosphorylation
GO:0006473	protein amino acid acetylation
GO:0006479	protein amino acid methylation
GO:0006605	protein targeting
GO:0006606	protein import into nucleus
GO:0006730	one-carbon compound metabolic process
GO:0006793	phosphorus metabolic process
GO:0006796	phosphate metabolic process
GO:0006879	cellular iron ion homeostasis
GO:0006886	intracellular protein transport
GO:0006913	nucleocytoplasmic transport
GO:0006950	response to stress
GO:0006974	response to DNA damage stimulus
GO:0006996	organelle organization and biogenesis
GO:0007010	cytoskeleton organization and biogenesis
GO:0007015	actin filament organization
GO:0007017	microtubule-based process
GO:0007020	microtubule nucleation
GO:0007049	cell cycle
GO:0007051	spindle organization and biogenesis
GO:0007056	female meiosis
GO:0007059	chromosome segregation
GO:0007062	sister chromatid cohesion
GO:0007067	M phase of mitotic cell cycle
GO:0007076	M phase of mitotic cell cycle
GO:0007088	regulation of mitosis
GO:0007093	mitotic cell cycle checkpoint
GO:0007094	mitotic cell cycle spindle assembly checkpoint
GO:0007098	centrosome cycle
GO:0007126	M phase of meiotic cell cycle
GO:0007127	M phase of meiotic cell cycle
GO:0007131	M phase of meiotic cell cycle
GO:0007143	female meiosis
GO:0007154	cell communication
GO:0007163	cell morphogenesis
GO:0007165	signal transduction
GO:0007166	cell surface receptor linked signal transduction
GO:0007167	enzyme linked receptor protein signaling pathway
GO:0007169	transmembrane receptor protein tyrosine kinase signaling pathway
GO:0007219	Notch signaling pathway
GO:0007242	intracellular signaling cascade
GO:0007264	small GTPase mediated signal transduction
GO:0007265	Ras protein signal transduction
GO:0007266	Rho protein signal transduction
GO:0007275	multicellular organismal development
GO:0007276	gamete generation
GO:0007280	pole cell migration
GO:0007281	germ cell development

GO:0007292	female gamete generation
GO:0007293	germarium-derived egg chamber formation
GO:0007294	germarium-derived oocyte fate determination
GO:0007304	ovarian follicle cell development
GO:0007306	ovarian follicle cell development
GO:0007307	ovarian follicle cell development
GO:0007346	regulation of progression through mitotic cell cycle
GO:0007389	multicellular organismal development
GO:0007399	nervous system development
GO:0008017	microtubule binding
GO:0008026	ATP-dependent helicase activity
GO:0008094	DNA-dependent ATPase activity
GO:0008104	protein localization
GO:0008134	transcription factor binding
GO:0008156	negative regulation of DNA replication
GO:0008168	methyltransferase activity
GO:0008170	N-methyltransferase activity
GO:0008213	protein amino acid alkylation
GO:0008234	cysteine-type peptidase activity
GO:0008276	protein methyltransferase activity
GO:0008283	cell proliferation
GO:0008296	3'-5'-exodeoxyribonuclease activity
GO:0008356	asymmetric cell division
GO:0008361	regulation of cell size
GO:0008378	galactosyltransferase activity
GO:0008380	RNA splicing
GO:0008757	S-adenosylmethionine-dependent methyltransferase activity
GO:0009057	macromolecule catabolic process
GO:0009653	anatomical structure development
GO:0009719	response to endogenous stimulus
GO:0009790	embryonic development
GO:0009791	multicellular organismal development
GO:0009798	axis specification
GO:0009889	regulation of biosynthetic process
GO:0009890	negative regulation of biosynthetic process
GO:0009892	negative regulation of metabolic process
GO:0009893	positive regulation of metabolic process
GO:0009948	anterior/posterior axis specification
GO:0009949	anterior/posterior axis specification
GO:0009950	dorsal/ventral axis specification
GO:0009951	dorsal/ventral axis specification
GO:0009952	anterior/posterior pattern formation
GO:0009953	dorsal/ventral pattern formation
GO:0009966	regulation of signal transduction
GO:0009994	oocyte differentiation
GO:0010467	gene expression
GO:0010468	regulation of gene expression
GO:0012505	endomembrane system
GO:0015031	protein transport
GO:0015630	microtubule cytoskeleton
GO:0015631	tubulin binding
GO:0016043	cellular component organization and biogenesis
GO:0016055	Wnt receptor signaling pathway
GO:0016070	RNA metabolic process
GO:0016071	mRNA metabolic process
GO:0016251	general RNA polymerase II transcription factor activity
GO:0016278	lysine N-methyltransferase activity
GO:0016279	protein-lysine N-methyltransferase activity
GO:0016301	kinase activity
GO:0016310	phosphorylation
GO:0016321	female meiosis
GO:0016333	morphogenesis of follicular epithelium

GO:0016358	generation of neurons
GO:0016455	RNA polymerase II transcription mediator activity
GO:0016458	gene silencing
GO:0016476	shape changes of embryonic cells
GO:0016481	negative regulation of transcription
GO:0016538	cyclin-dependent protein kinase regulator activity
GO:0016563	transcription activator activity
GO:0016564	transcription repressor activity
GO:0016568	chromatin modification
GO:0016569	covalent chromatin modification
GO:0016570	histone modification
GO:0016571	histone methylation
GO:0016572	histone phosphorylation
GO:0016573	histone acetylation
GO:0016585	chromatin remodeling complex
GO:0016591	DNA-directed RNA polymerase II, holoenzyme
GO:0016740	transferase activity
GO:0016741	transferase activity, transferring one-carbon groups
GO:0016772	transferase activity, transferring phosphorus-containing groups
GO:0016773	phosphotransferase activity, alcohol group as acceptor
GO:0016779	nucleotidyltransferase activity
GO:0016895	exodeoxyribonuclease activity, producing 5'-phosphomonoesters
GO:0017038	protein import
GO:0017124	SH3 domain binding
GO:0017145	stem cell division
GO:0017148	negative regulation of translation
GO:0018024	histone-lysine N-methyltransferase activity
GO:0018992	multicellular organismal development
GO:0019205	nucleobase, nucleoside, nucleotide kinase activity
GO:0019219	regulation of nucleobase, nucleoside, nucleotide and nucleic acid metabolic process
GO:0019220	regulation of phosphate metabolic process
GO:0019222	regulation of metabolic process
GO:0019783	small conjugating protein-specific protease activity
GO:0019899	enzyme binding
GO:0019904	protein domain specific binding
GO:0019953	sexual reproduction
GO:0022008	neurogenesis
GO:0022402	cell cycle
GO:0022403	cell cycle phase
GO:0022607	cellular component assembly
GO:0030030	cell morphogenesis
GO:0030032	lamellipodium biogenesis
GO:0030097	hemopoiesis
GO:0030154	cell differentiation
GO:0030182	generation of neurons
GO:0030237	multicellular organismal development
GO:0030261	chromosome condensation
GO:0030381	ovarian follicle cell development
GO:0030703	eggshell formation
GO:0030706	germarium-derived oocyte differentiation
GO:0030707	ovarian follicle cell development
GO:0030716	oocyte fate determination
GO:0030717	karyosome formation
GO:0030720	germarium-derived egg chamber formation
GO:0030727	germarium-derived female germ-line cyst formation
GO:0030894	replication fork
GO:0031023	microtubule organizing center organization and biogenesis
GO:0031056	regulation of histone modification
GO:0031123	RNA 3'-end processing
GO:0031124	mRNA 3'-end processing
GO:0031175	generation of neurons
GO:0031323	regulation of cellular metabolic process

GO:0031324	negative regulation of cellular metabolic process
GO:0031325	positive regulation of cellular metabolic process
GO:0031326	regulation of cellular biosynthetic process
GO:0031327	negative regulation of cellular biosynthetic process
GO:0031497	chromatin assembly
GO:0031519	PcG protein complex
GO:0031523	Myb complex
GO:0031577	spindle checkpoint
GO:0031965	nuclear envelope
GO:0031974	membrane-enclosed lumen
GO:0031981	nuclear lumen
GO:0032259	methylation
GO:0032502	developmental process
GO:0032774	RNA biosynthetic process
GO:0032837	distributive segregation
GO:0032989	cellular structure morphogenesis
GO:0032990	cell part morphogenesis
GO:0032991	macromolecular complex
GO:0033036	macromolecule localization
GO:0033301	cell cycle comprising mitosis without cytokinesis
GO:0035060	brahma complex
GO:0035102	PRC1 complex
GO:0035186	syncytial blastoderm mitotic cell cycle
GO:0035250	UDP-galactosyltransferase activity
GO:0035303	regulation of dephosphorylation
GO:0035304	regulation of protein amino acid dephosphorylation
GO:0040029	regulation of gene expression, epigenetic
GO:0042054	histone methyltransferase activity
GO:0042078	germ cell development
GO:0042575	DNA polymerase complex
GO:0042813	Wnt receptor activity
GO:0043138	3'-5' DNA helicase activity
GO:0043170	macromolecule metabolic process
GO:0043226	organelle
GO:0043227	membrane-bound organelle
GO:0043228	non-membrane-bound organelle
GO:0043229	intracellular organelle
GO:0043231	intracellular membrane-bound organelle
GO:0043232	intracellular non-membrane-bound organelle
GO:0043233	organelle lumen
GO:0043234	protein complex
GO:0043283	biopolymer metabolic process
GO:0043285	biopolymer catabolic process
GO:0043412	biopolymer modification
GO:0043414	biopolymer methylation
GO:0043543	protein amino acid acylation
GO:0043565	sequence-specific DNA binding
GO:0043596	nuclear replication fork
GO:0043601	nuclear replication fork
GO:0043623	cellular protein complex assembly
GO:0043687	post-translational protein modification
GO:0044237	cellular metabolic process
GO:0044238	primary metabolic process
GO:0044422	organelle
GO:0044424	intracellular
GO:0044427	chromosome
GO:0044428	nucleus
GO:0044430	cytoskeleton
GO:0044446	intracellular organelle
GO:0044450	microtubule organizing center
GO:0044451	nuclear lumen
GO:0044453	nuclear envelope

GO:0044454	nuclear chromosome
GO:0044464	cell
GO:0045132	M phase of meiotic cell cycle
GO:0045165	cell fate commitment
GO:0045184	establishment of protein localization
GO:0045448	mitotic cell cycle, embryonic
GO:0045449	regulation of transcription
GO:0045478	fusome organization and biogenesis
GO:0045786	negative regulation of progression through cell cycle
GO:0045892	negative regulation of transcription, DNA-dependent
GO:0045893	positive regulation of transcription, DNA-dependent
GO:0045934	negative regulation of nucleobase, nucleoside, nucleotide and nucleic acid metabolic process
GO:0045935	positive regulation of nucleobase, nucleoside, nucleotide and nucleic acid metabolic process
GO:0045941	positive regulation of transcription
GO:0045944	positive regulation of transcription from RNA polymerase II promoter
GO:0046907	intracellular transport
GO:0046974	histone lysine N-methyltransferase activity (H3-K9 specific)
GO:0048024	regulation of nuclear mRNA splicing, via spliceosome
GO:0048134	germ cell development
GO:0048135	female germ-line cyst formation
GO:0048468	cell development
GO:0048477	oogenesis
GO:0048513	organ development
GO:0048518	positive regulation of biological process
GO:0048519	negative regulation of biological process
GO:0048522	positive regulation of cellular process
GO:0048523	negative regulation of cellular process
GO:0048534	hemopoietic or lymphoid organ development
GO:0048646	anatomical structure development
GO:0048666	generation of neurons
GO:0048667	neuron morphogenesis during differentiation
GO:0048699	generation of neurons
GO:0048731	system development
GO:0048812	neuron morphogenesis during differentiation
GO:0048813	dendrite morphogenesis
GO:0048856	anatomical structure development
GO:0048858	cell morphogenesis
GO:0048869	cellular developmental process
GO:0050657	nucleic acid transport
GO:0050658	RNA transport
GO:0050684	regulation of mRNA processing
GO:0050789	regulation of biological process
GO:0050794	regulation of cellular process
GO:0051017	actin filament bundle formation
GO:0051052	regulation of DNA metabolic process
GO:0051053	negative regulation of DNA metabolic process
GO:0051128	regulation of cellular component organization and biogenesis
GO:0051168	nuclear export
GO:0051169	nuclear transport
GO:0051170	nuclear import
GO:0051174	regulation of phosphorus metabolic process
GO:0051179	localization
GO:0051225	spindle assembly
GO:0051234	establishment of localization
GO:0051236	establishment of RNA localization
GO:0051246	regulation of protein metabolic process
GO:0051248	negative regulation of protein metabolic process
GO:0051252	regulation of RNA metabolic process
GO:0051276	chromosome organization and biogenesis
GO:0051297	centrosome organization and biogenesis
GO:0051301	cell division
GO:0051320	interphase

GO:0051321	meiotic cell cycle
GO:0051322	anaphase
GO:0051325	interphase
GO:0051327	M phase of meiotic cell cycle
GO:0051329	interphase of mitotic cell cycle
GO:0051641	cellular localization
GO:0051649	cellular localization
GO:0051674	localization of cell
GO:0051726	regulation of cell cycle
GO:0055072	iron ion homeostasis
GO:0065001	specification of axis polarity
GO:0065003	macromolecular complex assembly
GO:0065004	protein-DNA complex assembly
GO:0065007	biological regulation
GO:0065009	regulation of a molecular function

Table S1E: Gene Ontologies Up-regulated in yw, w1118 DR and Canton-S DR. All gene ontologies have at least 5 genes in the GO and p value < 0.05.

GO:0000038	very-long-chain fatty acid metabolic process
GO:0000267	cell fraction
GO:0000902	cell morphogenesis
GO:0000904	cell development
GO:0001505	regulation of neurotransmitter levels
GO:0001736	establishment of planar polarity
GO:0001738	morphogenesis of a polarized epithelium
GO:0001751	compound eye photoreceptor cell differentiation
GO:0001752	compound eye photoreceptor fate commitment
GO:0001754	eye photoreceptor cell differentiation
GO:0002165	instar larval or pupal development
GO:0002376	immune system process
GO:0003008	system process
GO:0003779	actin binding
GO:0004035	alkaline phosphatase activity
GO:0004175	endopeptidase activity
GO:0004263	chymotrypsin activity
GO:0004723	calcium-dependent protein serine/threonine phosphatase activity
GO:0005102	receptor binding
GO:0005184	neuropeptide hormone activity
GO:0005200	structural constituent of cytoskeleton
GO:0005275	amine transmembrane transporter activity
GO:0005283	sodium:amino acid symporter activity
GO:0005328	neurotransmitter:sodium symporter activity
GO:0005342	organic acid transmembrane transporter activity
GO:0005416	cation:amino acid symporter activity
GO:0005509	calcium ion binding
GO:0005515	protein binding
GO:0005516	calmodulin binding
GO:0005578	proteinaceous extracellular matrix
GO:0005604	basement membrane
GO:0005624	membrane fraction
GO:0005792	microsome
GO:0005859	muscle myosin complex
GO:0005865	sarcomere
GO:0005886	plasma membrane
GO:0005887	integral to plasma membrane
GO:0006810	transport
GO:0006811	ion transport
GO:0006812	cation transport
GO:0006817	phosphate transport
GO:0006820	anion transport

GO:0006874	cellular calcium ion homeostasis
GO:0006928	cell motility
GO:0006952	defense response
GO:0006955	immune response
GO:0007154	cell communication
GO:0007155	cell adhesion
GO:0007164	establishment of tissue polarity
GO:0007165	signal transduction
GO:0007166	cell surface receptor linked signal transduction
GO:0007167	enzyme linked receptor protein signaling pathway
GO:0007169	transmembrane receptor protein tyrosine kinase signaling pathway
GO:0007186	G-protein coupled receptor protein signaling pathway
GO:0007200	G-protein signaling, coupled to IP3 second messenger (phospholipase C activating)
GO:0007218	neuropeptide signaling pathway
GO:0007242	intracellular signaling cascade
GO:0007267	cell-cell signaling
GO:0007268	synaptic transmission
GO:0007275	multicellular organismal development
GO:0007399	nervous system development
GO:0007409	axonogenesis
GO:0007411	axonogenesis
GO:0007416	synaptogenesis
GO:0007424	open tracheal system development
GO:0007431	salivary gland development
GO:0007432	salivary gland boundary specification
GO:0007439	ectodermal gut development
GO:0007447	imaginal disc development
GO:0007472	wing disc morphogenesis
GO:0007473	wing disc proximal/distal pattern formation
GO:0007476	imaginal disc-derived wing morphogenesis
GO:0007478	leg disc morphogenesis
GO:0007498	mesoderm development
GO:0007507	heart development
GO:0007517	muscle development
GO:0007519	skeletal muscle development
GO:0007525	somatic muscle development
GO:0007528	skeletal muscle fiber development
GO:0007548	sex differentiation
GO:0007601	visual perception
GO:0007602	phototransduction
GO:0007623	circadian rhythm
GO:0008020	G-protein coupled photoreceptor activity
GO:0008083	growth factor activity
GO:0008084	imaginal disc growth factor activity
GO:0008092	cytoskeletal protein binding
GO:0008253	5'-nucleotidase activity
GO:0008271	secondary active sulfate transmembrane transporter activity
GO:0008277	regulation of G-protein coupled receptor protein signaling pathway
GO:0008324	cation transmembrane transporter activity
GO:0008507	sodium:iodide symporter activity
GO:0008509	anion transmembrane transporter activity
GO:0008543	fibroblast growth factor receptor signaling pathway
GO:0008586	imaginal disc-derived wing morphogenesis
GO:0009309	amine biosynthetic process
GO:0009314	response to radiation
GO:0009416	response to light stimulus
GO:0009581	detection of external stimulus
GO:0009582	detection of abiotic stimulus
GO:0009583	detection of light stimulus
GO:0009584	detection of visible light
GO:0009586	rhodopsin mediated phototransduction
GO:0009595	detection of biotic stimulus

GO:0009605	response to external stimulus
GO:0009607	response to biotic stimulus
GO:0009617	response to bacterium
GO:0009628	response to abiotic stimulus
GO:0009791	multicellular organismal development
GO:0009881	photoreceptor activity
GO:0009888	organ development
GO:0014706	striated muscle development
GO:0015075	ion transmembrane transporter activity
GO:0015101	organic cation transmembrane transporter activity
GO:0015116	sulfate transmembrane transporter activity
GO:0015179	L-amino acid transmembrane transporter activity
GO:0015291	secondary active transmembrane transporter activity
GO:0015293	symporter activity
GO:0015294	solute:cation symporter activity
GO:0015296	anion:cation symporter activity
GO:0015370	solute:sodium symporter activity
GO:0015373	monovalent anion:sodium symporter activity
GO:0015381	high affinity sulfate transmembrane transporter activity
GO:0015629	actin cytoskeleton
GO:0015698	inorganic anion transport
GO:0016020	membrane
GO:0016021	integral to membrane
GO:0016023	cytoplasmic membrane-bound vesicle
GO:0016027	rhabdomere
GO:0016028	rhabdomere
GO:0016045	detection of bacterium
GO:0016059	deactivation of rhodopsin mediated signaling
GO:0016459	actin cytoskeleton
GO:0016460	myosin II complex
GO:0016477	cell migration
GO:0016511	endothelin-converting enzyme activity
GO:0016787	hydrolase activity
GO:0016791	phosphoric monoester hydrolase activity
GO:0016810	hydrolase activity, acting on carbon-nitrogen (but not peptide) bonds
GO:0016811	hydrolase activity, acting on carbon-nitrogen (but not peptide) bonds, in linear amides
GO:0019226	transmission of nerve impulse
GO:0019722	calcium-mediated signaling
GO:0019897	extrinsic to plasma membrane
GO:0019898	extrinsic to membrane
GO:0019932	second-messenger-mediated signaling
GO:0021700	developmental maturation
GO:0022008	neurogenesis
GO:0022610	biological adhesion
GO:0022804	active transmembrane transporter activity
GO:0022857	transmembrane transporter activity
GO:0022891	substrate-specific transmembrane transporter activity
GO:0022892	substrate-specific transporter activity
GO:0030016	myofibril
GO:0030017	sarcomere
GO:0030029	actin filament-based process
GO:0030030	cell morphogenesis
GO:0030036	actin cytoskeleton organization and biogenesis
GO:0030154	cell differentiation
GO:0030182	generation of neurons
GO:0030239	myoblast maturation
GO:0031012	extracellular matrix
GO:0031175	generation of neurons
GO:0031224	intrinsic to membrane
GO:0031226	intrinsic to plasma membrane
GO:0031410	cytoplasmic vesicle
GO:0031982	vesicle

GO:0031988	membrane-bound vesicle
GO:0032501	multicellular organismal process
GO:0032989	cellular structure morphogenesis
GO:0032990	cell part morphogenesis
GO:0035107	appendage morphogenesis
GO:0035114	imaginal disc-derived appendage morphogenesis
GO:0035152	regulation of tube architecture, open tracheal system
GO:0035153	epithelial cell type specification, open tracheal system
GO:0035215	genital disc development
GO:0035216	haltere disc development
GO:0035218	leg disc development
GO:0035220	wing disc development
GO:0035222	wing disc pattern formation
GO:0035272	exocrine system development
GO:0035277	spiracle morphogenesis, open tracheal system
GO:0042133	regulation of neurotransmitter levels
GO:0042136	neurotransmitter biosynthetic process
GO:0042330	taxis
GO:0042398	amino acid derivative biosynthetic process
GO:0042445	hormone metabolic process
GO:0042578	phosphoric ester hydrolase activity
GO:0042592	homeostatic process
GO:0042598	vesicular fraction
GO:0042692	muscle cell differentiation
GO:0042706	eye photoreceptor cell fate commitment
GO:0042742	defense response to bacterium
GO:0042995	cell projection
GO:0043167	ion binding
GO:0043169	cation binding
GO:0043190	ATP-binding cassette (ABC) transporter complex
GO:0043292	contractile fiber
GO:0044271	nitrogen compound biosynthetic process
GO:0044420	extracellular matrix
GO:0044421	extracellular region
GO:0044425	membrane
GO:0044449	contractile fiber
GO:0044459	plasma membrane part
GO:0045137	multicellular organismal development
GO:0045165	cell fate commitment
GO:0045211	postsynaptic membrane
GO:0045445	skeletal muscle fiber development
GO:0046530	photoreceptor cell differentiation
GO:0046552	photoreceptor cell fate commitment
GO:0046661	multicellular organismal development
GO:0046872	metal ion binding
GO:0046943	carboxylic acid transmembrane transporter activity
GO:0046983	protein dimerization activity
GO:0048015	phosphoinositide-mediated signaling
GO:0048468	cell development
GO:0048469	cell development
GO:0048511	rhythmic process
GO:0048512	circadian behavior
GO:0048513	organ development
GO:0048565	gut development
GO:0048567	ectodermal gut morphogenesis
GO:0048627	skeletal muscle fiber development
GO:0048628	myoblast maturation
GO:0048666	generation of neurons
GO:0048667	neuron morphogenesis during differentiation
GO:0048699	generation of neurons
GO:0048731	system development
GO:0048732	gland development

GO:0048736	appendage development
GO:0048737	imaginal disc-derived appendage development
GO:0048741	skeletal muscle fiber development
GO:0048747	muscle fiber development
GO:0048812	neuron morphogenesis during differentiation
GO:0048856	anatomical structure development
GO:0048858	cell morphogenesis
GO:0048869	cellular developmental process
GO:0050789	regulation of biological process
GO:0050790	regulation of catalytic activity
GO:0050793	regulation of developmental process
GO:0050808	synapse organization and biogenesis
GO:0050877	neurological system process
GO:0050896	response to stimulus
GO:0050906	detection of stimulus during sensory perception
GO:0050908	detection of light stimulus during visual perception
GO:0050953	sensory perception of light stimulus
GO:0050962	detection of light stimulus during sensory perception
GO:0051146	striated muscle cell differentiation
GO:0051179	localization
GO:0051234	establishment of localization
GO:0051606	detection of stimulus
GO:0051674	localization of cell
GO:0051704	multi-organism process
GO:0051707	response to other organism
GO:0055001	muscle cell development
GO:0055002	striated muscle cell development
GO:0055074	calcium ion homeostasis
GO:0065007	biological regulation
GO:0065008	regulation of biological quality
GO:0065009	regulation of a molecular function

Table S1F: Gene Ontologies Down-regulated in yw, w1118 DR and Canton-S DR. All gene ontologies have at least 5 genes in the GO and p value < 0.05.

GO:0000070	M phase of mitotic cell cycle
GO:0000074	regulation of progression through cell cycle
GO:0000075	cell cycle checkpoint
GO:0000082	interphase of mitotic cell cycle
GO:0000084	interphase of mitotic cell cycle
GO:0000087	M phase of mitotic cell cycle
GO:0000119	DNA-directed RNA polymerase II, holoenzyme
GO:0000122	negative regulation of transcription from RNA polymerase II promoter
GO:0000226	microtubule cytoskeleton organization and biogenesis
GO:0000228	nuclear chromosome
GO:0000278	mitotic cell cycle
GO:0000279	M phase
GO:0000375	RNA splicing, via transesterification reactions
GO:0000377	RNA splicing, via transesterification reactions with bulged adenosine as nucleophile
GO:0000380	alternative nuclear mRNA splicing, via spliceosome
GO:0000381	regulation of alternative nuclear mRNA splicing, via spliceosome
GO:0000398	nuclear mRNA splicing, via spliceosome
GO:0000775	chromosome, pericentric region
GO:0000776	chromosome, pericentric region
GO:0000777	condensed chromosome, pericentric region
GO:0000779	condensed chromosome, pericentric region
GO:0000785	chromatin
GO:0000792	heterochromatin
GO:0000793	condensed chromosome
GO:0000808	origin recognition complex
GO:0000819	sister chromatid segregation

GO:0000910	cytokinesis
GO:0000940	condensed chromosome, pericentric region
GO:0003002	regionalization
GO:0003676	nucleic acid binding
GO:0003677	DNA binding
GO:0003678	DNA helicase activity
GO:0003682	chromatin binding
GO:0003702	RNA polymerase II transcription factor activity
GO:0003712	transcription cofactor activity
GO:0003713	transcription coactivator activity
GO:0003723	RNA binding
GO:0003729	mRNA binding
GO:0003777	microtubule motor activity
GO:0003887	DNA-directed DNA polymerase activity
GO:0004003	ATP-dependent DNA helicase activity
GO:0004386	helicase activity
GO:0004518	nuclease activity
GO:0004519	endonuclease activity
GO:0004520	endodeoxyribonuclease activity
GO:0004529	exodeoxyribonuclease activity
GO:0004536	deoxyribonuclease activity
GO:0004672	protein kinase activity
GO:0004674	protein serine/threonine kinase activity
GO:0005515	protein binding
GO:0005622	intracellular
GO:0005623	cell
GO:0005634	nucleus
GO:0005635	nuclear envelope
GO:0005654	nuclear lumen
GO:0005656	pre-replicative complex
GO:0005657	replication fork
GO:0005663	DNA replication factor C complex
GO:0005664	nuclear origin of replication recognition complex
GO:0005667	transcription factor complex
GO:0005669	DNA-directed RNA polymerase II, holoenzyme
GO:0005678	chromatin assembly complex
GO:0005694	chromosome
GO:0005700	polytene chromosome
GO:0005813	centrosome
GO:0005815	microtubule organizing center
GO:0005819	spindle
GO:0005828	kinetochore microtubule
GO:0005856	cytoskeleton
GO:0005871	kinesin complex
GO:0005874	microtubule
GO:0005876	spindle microtubule
GO:0006139	nucleobase, nucleoside, nucleotide and nucleic acid metabolic process
GO:0006259	DNA metabolic process
GO:0006260	DNA replication
GO:0006261	DNA-dependent DNA replication
GO:0006267	pre-replicative complex assembly
GO:0006270	DNA-dependent DNA replication
GO:0006275	regulation of DNA replication
GO:0006277	DNA amplification
GO:0006281	DNA repair
GO:0006289	nucleotide-excision repair
GO:0006310	DNA recombination
GO:0006334	nucleosome assembly
GO:0006350	transcription
GO:0006351	transcription, DNA-dependent
GO:0006352	transcription initiation
GO:0006355	regulation of transcription, DNA-dependent

GO:0006357	regulation of transcription from RNA polymerase II promoter
GO:0006366	transcription from RNA polymerase II promoter
GO:0006367	transcription initiation from RNA polymerase II promoter
GO:0006396	RNA processing
GO:0006397	mRNA processing
GO:0006464	protein modification process
GO:0006468	protein amino acid phosphorylation
GO:0006470	protein amino acid dephosphorylation
GO:0006606	protein import into nucleus
GO:0006793	phosphorus metabolic process
GO:0006796	phosphate metabolic process
GO:0006950	response to stress
GO:0006974	response to DNA damage stimulus
GO:0006996	organelle organization and biogenesis
GO:0007010	cytoskeleton organization and biogenesis
GO:0007017	microtubule-based process
GO:0007049	cell cycle
GO:0007051	spindle organization and biogenesis
GO:0007059	chromosome segregation
GO:0007067	M phase of mitotic cell cycle
GO:0007076	M phase of mitotic cell cycle
GO:0007088	regulation of mitosis
GO:0007093	mitotic cell cycle checkpoint
GO:0007094	mitotic cell cycle spindle assembly checkpoint
GO:0007098	centrosome cycle
GO:0007126	M phase of meiotic cell cycle
GO:0007127	M phase of meiotic cell cycle
GO:0007131	M phase of meiotic cell cycle
GO:0007143	female meiosis
GO:0007275	multicellular organismal development
GO:0007276	gamete generation
GO:0007280	pole cell migration
GO:0007281	germ cell development
GO:0007292	female gamete generation
GO:0007293	germarium-derived egg chamber formation
GO:0007294	germarium-derived oocyte fate determination
GO:0007304	ovarian follicle cell development
GO:0007306	ovarian follicle cell development
GO:0007307	ovarian follicle cell development
GO:0007346	regulation of progression through mitotic cell cycle
GO:0007389	multicellular organismal development
GO:0008026	ATP-dependent helicase activity
GO:0008094	DNA-dependent ATPase activity
GO:0008134	transcription factor binding
GO:0008156	negative regulation of DNA replication
GO:0008168	methyltransferase activity
GO:0008276	protein methyltransferase activity
GO:0008283	cell proliferation
GO:0008296	3'-5'-exodeoxyribonuclease activity
GO:0008356	asymmetric cell division
GO:0008380	RNA splicing
GO:0009653	anatomical structure development
GO:0009719	response to endogenous stimulus
GO:0009790	embryonic development
GO:0009798	axis specification
GO:0009892	negative regulation of metabolic process
GO:0009893	positive regulation of metabolic process
GO:0009948	anterior/posterior axis specification
GO:0009949	anterior/posterior axis specification
GO:0009950	dorsal/ventral axis specification
GO:0009951	dorsal/ventral axis specification
GO:0009952	anterior/posterior pattern formation

GO:0009953	dorsal/ventral pattern formation
GO:0009994	oocyte differentiation
GO:0010467	gene expression
GO:0010468	regulation of gene expression
GO:0015630	microtubule cytoskeleton
GO:0016043	cellular component organization and biogenesis
GO:0016070	RNA metabolic process
GO:0016071	mRNA metabolic process
GO:0016251	general RNA polymerase II transcription factor activity
GO:0016301	kinase activity
GO:0016310	phosphorylation
GO:0016321	female meiosis
GO:0016455	RNA polymerase II transcription mediator activity
GO:0016481	negative regulation of transcription
GO:0016538	cyclin-dependent protein kinase regulator activity
GO:0016563	transcription activator activity
GO:0016564	transcription repressor activity
GO:0016568	chromatin modification
GO:0016569	covalent chromatin modification
GO:0016570	histone modification
GO:0016572	histone phosphorylation
GO:0016591	DNA-directed RNA polymerase II, holoenzyme
GO:0016740	transferase activity
GO:0016772	transferase activity, transferring phosphorus-containing groups
GO:0016773	phosphotransferase activity, alcohol group as acceptor
GO:0016779	nucleotidyltransferase activity
GO:0016895	exodeoxyribonuclease activity, producing 5'-phosphomonoesters
GO:0017038	protein import
GO:0019219	regulation of nucleobase, nucleoside, nucleotide and nucleic acid metabolic process
GO:0019220	regulation of phosphate metabolic process
GO:0019222	regulation of metabolic process
GO:0019953	sexual reproduction
GO:0022402	cell cycle
GO:0022403	cell cycle phase
GO:0022607	cellular component assembly
GO:0030154	cell differentiation
GO:0030261	chromosome condensation
GO:0030703	eggshell formation
GO:0030706	germarium-derived oocyte differentiation
GO:0030707	ovarian follicle cell development
GO:0030716	oocyte fate determination
GO:0030717	karyosome formation
GO:0030720	germarium-derived egg chamber formation
GO:0030727	germarium-derived female germ-line cyst formation
GO:0030894	replication fork
GO:0031023	microtubule organizing center organization and biogenesis
GO:0031323	regulation of cellular metabolic process
GO:0031324	negative regulation of cellular metabolic process
GO:0031325	positive regulation of cellular metabolic process
GO:0031519	PcG protein complex
GO:0031523	Myb complex
GO:0031577	spindle checkpoint
GO:0031965	nuclear envelope
GO:0031974	membrane-enclosed lumen
GO:0031981	nuclear lumen
GO:0032502	developmental process
GO:0032774	RNA biosynthetic process
GO:0032837	distributive segregation
GO:0032991	macromolecular complex
GO:0033036	macromolecule localization
GO:0033301	cell cycle comprising mitosis without cytokinesis
GO:0035102	PRC1 complex

GO:0035186	syncytial blastoderm mitotic cell cycle
GO:0035303	regulation of dephosphorylation
GO:0035304	regulation of protein amino acid dephosphorylation
GO:0042054	histone methyltransferase activity
GO:0042575	DNA polymerase complex
GO:0043138	3'-5' DNA helicase activity
GO:0043170	macromolecule metabolic process
GO:0043226	organelle
GO:0043227	membrane-bound organelle
GO:0043228	non-membrane-bound organelle
GO:0043229	intracellular organelle
GO:0043231	intracellular membrane-bound organelle
GO:0043232	intracellular non-membrane-bound organelle
GO:0043233	organelle lumen
GO:0043234	protein complex
GO:0043283	biopolymer metabolic process
GO:0043412	biopolymer modification
GO:0043414	biopolymer methylation
GO:0043596	nuclear replication fork
GO:0043601	nuclear replication fork
GO:0043623	cellular protein complex assembly
GO:0043687	post-translational protein modification
GO:0044237	cellular metabolic process
GO:0044238	primary metabolic process
GO:0044422	organelle
GO:0044424	intracellular
GO:0044427	chromosome
GO:0044428	nucleus
GO:0044430	cytoskeleton
GO:0044446	intracellular organelle
GO:0044450	microtubule organizing center
GO:0044451	nuclear lumen
GO:0044453	nuclear envelope
GO:0044454	nuclear chromosome
GO:0044464	cell
GO:0045132	M phase of meiotic cell cycle
GO:0045448	mitotic cell cycle, embryonic
GO:0045449	regulation of transcription
GO:0045786	negative regulation of progression through cell cycle
GO:0045892	negative regulation of transcription, DNA-dependent
GO:0045893	positive regulation of transcription, DNA-dependent
GO:0045934	negative regulation of nucleobase, nucleoside, nucleotide and nucleic acid metabolic process
GO:0045935	positive regulation of nucleobase, nucleoside, nucleotide and nucleic acid metabolic process
GO:0045941	positive regulation of transcription
GO:0046974	histone lysine N-methyltransferase activity (H3-K9 specific)
GO:0048024	regulation of nuclear mRNA splicing, via spliceosome
GO:0048134	germ cell development
GO:0048135	female germ-line cyst formation
GO:0048468	cell development
GO:0048477	oogenesis
GO:0048518	positive regulation of biological process
GO:0048519	negative regulation of biological process
GO:0048522	positive regulation of cellular process
GO:0048523	negative regulation of cellular process
GO:0048646	anatomical structure development
GO:0048856	anatomical structure development
GO:0048869	cellular developmental process
GO:0050684	regulation of mRNA processing
GO:0050789	regulation of biological process
GO:0050794	regulation of cellular process
GO:0051052	regulation of DNA metabolic process
GO:0051053	negative regulation of DNA metabolic process

GO:0051170	nuclear import
GO:0051174	regulation of phosphorus metabolic process
GO:0051225	spindle assembly
GO:0051246	regulation of protein metabolic process
GO:0051248	negative regulation of protein metabolic process
GO:0051252	regulation of RNA metabolic process
GO:0051276	chromosome organization and biogenesis
GO:0051297	centrosome organization and biogenesis
GO:0051301	cell division
GO:0051320	interphase
GO:0051321	meiotic cell cycle
GO:0051322	anaphase
GO:0051325	interphase
GO:0051327	M phase of meiotic cell cycle
GO:0051329	interphase of mitotic cell cycle
GO:0051726	regulation of cell cycle
GO:0065001	specification of axis polarity
GO:0065003	macromolecular complex assembly
GO:0065004	protein-DNA complex assembly
GO:0065007	biological regulation

Table S1G: Gene Ontologies Up-regulated in dSir2 overexpression. All gene ontologies have at least 5 genes in the GO and p value < 0.05.

GO:0000267	cell fraction
GO:0003012	muscle system process
GO:0003779	actin binding
GO:0004175	endopeptidase activity
GO:0004252	serine-type endopeptidase activity
GO:0004857	enzyme inhibitor activity
GO:0004866	endopeptidase inhibitor activity
GO:0005200	structural constituent of cytoskeleton
GO:0005372	water transporter activity
GO:0005388	calcium-transporting ATPase activity
GO:0005509	calcium ion binding
GO:0005578	proteinaceous extracellular matrix
GO:0005604	basement membrane
GO:0005624	membrane fraction
GO:0005792	microsome
GO:0005856	cytoskeleton
GO:0005859	muscle myosin complex
GO:0005865	sarcomere
GO:0006629	lipid metabolic process
GO:0006816	calcium ion transport
GO:0006874	cellular calcium ion homeostasis
GO:0006875	cellular metal ion homeostasis
GO:0006936	muscle contraction
GO:0006952	defense response
GO:0007498	mesoderm development
GO:0007519	skeletal muscle development
GO:0007601	visual perception
GO:0007602	phototransduction
GO:0007604	phototransduction, UV
GO:0008233	peptidase activity
GO:0008236	serine-type peptidase activity
GO:0008238	exopeptidase activity
GO:0008252	nucleotidase activity
GO:0008253	5'-nucleotidase activity
GO:0009055	electron carrier activity
GO:0009314	response to radiation
GO:0009411	response to UV

GO:0009416	response to light stimulus
GO:0009581	detection of external stimulus
GO:0009582	detection of abiotic stimulus
GO:0009583	detection of light stimulus
GO:0009584	detection of visible light
GO:0009589	detection of UV
GO:0009605	response to external stimulus
GO:0009636	response to toxin
GO:0009888	organ development
GO:0009968	negative regulation of signal transduction
GO:0014706	striated muscle development
GO:0015020	glucuronosyltransferase activity
GO:0015085	calcium ion transmembrane transporter activity
GO:0015291	secondary active transmembrane transporter activity
GO:0015629	actin cytoskeleton
GO:0015674	di-, tri-valent inorganic cation transport
GO:0016028	rhabdomere
GO:0016059	deactivation of rhodopsin mediated signaling
GO:0016460	myosin II complex
GO:0016491	oxidoreductase activity
GO:0016705	oxidoreductase activity, acting on paired donors, with incorporation or reduction of molecular oxygen
GO:0016787	hydrolase activity
GO:0016791	phosphoric monoester hydrolase activity
GO:0017171	serine hydrolase activity
GO:0019203	carbohydrate phosphatase activity
GO:0019725	cellular homeostasis
GO:0019829	cation-transporting ATPase activity
GO:0022804	active transmembrane transporter activity
GO:0022857	transmembrane transporter activity
GO:0022892	substrate-specific transporter activity
GO:0030016	myofibril
GO:0030017	sarcomere
GO:0030239	myoblast maturation
GO:0030414	protease inhibitor activity
GO:0031012	extracellular matrix
GO:0042578	phosphoric ester hydrolase activity
GO:0042592	homeostatic process
GO:0042598	vesicular fraction
GO:0042692	muscle cell differentiation
GO:0042708	elastase activity
GO:0043167	ion binding
GO:0043169	cation binding
GO:0043292	contractile fiber
GO:0044420	extracellular matrix
GO:0044421	extracellular region
GO:0044430	cytoskeleton
GO:0044449	contractile fiber
GO:0045445	skeletal muscle fiber development
GO:0046872	metal ion binding
GO:0048469	cell development
GO:0048627	skeletal muscle fiber development
GO:0048628	myoblast maturation
GO:0050906	detection of stimulus during sensory perception
GO:0050908	detection of light stimulus during visual perception
GO:0050953	sensory perception of light stimulus
GO:0050962	detection of light stimulus during sensory perception
GO:0051146	striated muscle cell differentiation
GO:0051606	detection of stimulus
GO:0055001	muscle cell development
GO:0055002	striated muscle cell development
GO:0055065	metal ion homeostasis
GO:0055074	calcium ion homeostasis

Table S1H: Gene Ontologies Down-regulated in dSir2 overexpression. All gene ontologies have at least 5 genes in the GO and p value < 0.05.

GO:0000070	M phase of mitotic cell cycle
GO:0000074	regulation of progression through cell cycle
GO:0000079	regulation of cyclin-dependent protein kinase activity
GO:0000082	interphase of mitotic cell cycle
GO:0000087	M phase of mitotic cell cycle
GO:0000228	nuclear chromosome
GO:0000278	mitotic cell cycle
GO:0000279	M phase
GO:0000578	embryonic axis specification
GO:0000775	chromosome, pericentric region
GO:0000785	chromatin
GO:0000792	heterochromatin
GO:0000793	condensed chromosome
GO:0000794	condensed nuclear chromosome
GO:0000808	origin recognition complex
GO:0000819	sister chromatid segregation
GO:0000902	cell morphogenesis
GO:0000910	cytokinesis
GO:0001709	cell fate commitment
GO:0003676	nucleic acid binding
GO:0003677	DNA binding
GO:0003682	chromatin binding
GO:0004437	inositol or phosphatidylinositol phosphatase activity
GO:0004672	protein kinase activity
GO:0004674	protein serine/threonine kinase activity
GO:0005083	small GTPase regulator activity
GO:0005515	protein binding
GO:0005616	extracellular space
GO:0005622	intracellular
GO:0005623	cell
GO:0005634	nucleus
GO:0005654	nuclear lumen
GO:0005664	nuclear origin of replication recognition complex
GO:0005667	transcription factor complex
GO:0005694	chromosome
GO:0005700	polytene chromosome
GO:0005720	nuclear heterochromatin
GO:0005725	intercalary heterochromatin
GO:0005819	spindle
GO:0005856	cytoskeleton
GO:0005938	cell cortex
GO:0006139	nucleobase, nucleoside, nucleotide and nucleic acid metabolic process
GO:0006259	DNA metabolic process
GO:0006260	DNA replication
GO:0006261	DNA-dependent DNA replication
GO:0006281	DNA repair
GO:0006298	mismatch repair
GO:0006323	DNA packaging
GO:0006325	establishment and/or maintenance of chromatin architecture
GO:0006350	transcription
GO:0006351	transcription, DNA-dependent
GO:0006355	regulation of transcription, DNA-dependent
GO:0006357	regulation of transcription from RNA polymerase II promoter
GO:0006366	transcription from RNA polymerase II promoter
GO:0006396	RNA processing
GO:0006464	protein modification process
GO:0006468	protein amino acid phosphorylation

GO:0006793	phosphorus metabolic process
GO:0006796	phosphate metabolic process
GO:0006950	response to stress
GO:0006974	response to DNA damage stimulus
GO:0006996	organelle organization and biogenesis
GO:0007049	cell cycle
GO:0007059	chromosome segregation
GO:0007067	M phase of mitotic cell cycle
GO:0007076	M phase of mitotic cell cycle
GO:0007088	regulation of mitosis
GO:0007143	female meiosis
GO:0007154	cell communication
GO:0007165	signal transduction
GO:0007166	cell surface receptor linked signal transduction
GO:0007167	enzyme linked receptor protein signaling pathway
GO:0007169	transmembrane receptor protein tyrosine kinase signaling pathway
GO:0007242	intracellular signaling cascade
GO:0007264	small GTPase mediated signal transduction
GO:0007265	Ras protein signal transduction
GO:0007275	multicellular organismal development
GO:0007276	gamete generation
GO:0007292	female gamete generation
GO:0007293	germarium-derived egg chamber formation
GO:0007350	blastoderm segmentation
GO:0007351	blastoderm segmentation
GO:0007399	nervous system development
GO:0008283	cell proliferation
GO:0008293	torso signaling pathway
GO:0008356	asymmetric cell division
GO:0008360	regulation of cell shape
GO:0008375	acetylglucosaminyltransferase activity
GO:0008595	blastoderm segmentation
GO:0009653	anatomical structure development
GO:0009719	response to endogenous stimulus
GO:0009790	embryonic development
GO:0009880	embryonic development
GO:0009892	negative regulation of metabolic process
GO:0009893	positive regulation of metabolic process
GO:0010467	gene expression
GO:0010468	regulation of gene expression
GO:0015630	microtubule cytoskeleton
GO:0016043	cellular component organization and biogenesis
GO:0016070	RNA metabolic process
GO:0016301	kinase activity
GO:0016310	phosphorylation
GO:0016476	shape changes of embryonic cells
GO:0016481	negative regulation of transcription
GO:0016563	transcription activator activity
GO:0016568	chromatin modification
GO:0016572	histone phosphorylation
GO:0016740	transferase activity
GO:0016772	transferase activity, transferring phosphorus-containing groups
GO:0016773	phosphotransferase activity, alcohol group as acceptor
GO:0017145	stem cell division
GO:0019219	regulation of nucleobase, nucleoside, nucleotide and nucleic acid metabolic process
GO:0019222	regulation of metabolic process
GO:0019953	sexual reproduction
GO:0019992	diacylglycerol binding
GO:0022008	neurogenesis
GO:0022402	cell cycle
GO:0022403	cell cycle phase
GO:0022603	regulation of anatomical structure morphogenesis

GO:0022604	regulation of cell morphogenesis
GO:0030154	cell differentiation
GO:0030182	generation of neurons
GO:0030496	midbody
GO:0030695	GTPase regulator activity
GO:0030727	germarium-derived female germ-line cyst formation
GO:0031323	regulation of cellular metabolic process
GO:0031324	negative regulation of cellular metabolic process
GO:0031325	positive regulation of cellular metabolic process
GO:0031497	chromatin assembly
GO:0031519	PcG protein complex
GO:0032502	developmental process
GO:0032774	RNA biosynthetic process
GO:0032837	distributive segregation
GO:0032989	cellular structure morphogenesis
GO:0033301	cell cycle comprising mitosis without cytokinesis
GO:0035102	PRC1 complex
GO:0035186	syncytial blastoderm mitotic cell cycle
GO:0035282	segmentation
GO:0042078	germ cell development
GO:0043170	macromolecule metabolic process
GO:0043226	organelle
GO:0043227	membrane-bound organelle
GO:0043228	non-membrane-bound organelle
GO:0043229	intracellular organelle
GO:0043231	intracellular membrane-bound organelle
GO:0043232	intracellular non-membrane-bound organelle
GO:0043234	protein complex
GO:0043283	biopolymer metabolic process
GO:0043412	biopolymer modification
GO:0043687	post-translational protein modification
GO:0044237	cellular metabolic process
GO:0044238	primary metabolic process
GO:0044422	organelle
GO:0044424	intracellular
GO:0044427	chromosome
GO:0044428	nucleus
GO:0044430	cytoskeleton
GO:0044446	intracellular organelle
GO:0044451	nuclear lumen
GO:0044454	nuclear chromosome
GO:0044464	cell
GO:0045005	DNA-dependent DNA replication
GO:0045448	mitotic cell cycle, embryonic
GO:0045449	regulation of transcription
GO:0045786	negative regulation of progression through cell cycle
GO:0045892	negative regulation of transcription, DNA-dependent
GO:0045935	positive regulation of nucleobase, nucleoside, nucleotide and nucleic acid metabolic process
GO:0045941	positive regulation of transcription
GO:0048134	germ cell development
GO:0048135	female germ-line cyst formation
GO:0048477	oogenesis
GO:0048513	organ development
GO:0048518	positive regulation of biological process
GO:0048519	negative regulation of biological process
GO:0048522	positive regulation of cellular process
GO:0048523	negative regulation of cellular process
GO:0048646	anatomical structure development
GO:0048699	generation of neurons
GO:0048731	system development
GO:0048856	anatomical structure development
GO:0048869	cellular developmental process

GO:0050789	regulation of biological process
GO:0050793	regulation of developmental process
GO:0050794	regulation of cellular process
GO:0051276	chromosome organization and biogenesis
GO:0051301	cell division
GO:0051325	interphase
GO:0051329	interphase of mitotic cell cycle
GO:0051726	regulation of cell cycle
GO:0065007	biological regulation

Table S1I: Gene Ontologies Up-regulated in yw, w1118 DR and dSir2 overexpression All gene ontologies have at least 5 genes in the GO and p value < 0.05.

GO:0000267	cell fraction
GO:0003779	actin binding
GO:0004175	endopeptidase activity
GO:0004252	serine-type endopeptidase activity
GO:0005200	structural constituent of cytoskeleton
GO:0005509	calcium ion binding
GO:0005578	proteinaceous extracellular matrix
GO:0005604	basement membrane
GO:0005624	membrane fraction
GO:0005792	microsome
GO:0005859	muscle myosin complex
GO:0005865	sarcomere
GO:0006629	lipid metabolic process
GO:0006874	cellular calcium ion homeostasis
GO:0006952	defense response
GO:0007498	mesoderm development
GO:0007519	skeletal muscle development
GO:0007601	visual perception
GO:0007602	phototransduction
GO:0008233	peptidase activity
GO:0008236	serine-type peptidase activity
GO:0008238	exopeptidase activity
GO:0008253	5'-nucleotidase activity
GO:0009314	response to radiation
GO:0009416	response to light stimulus
GO:0009581	detection of external stimulus
GO:0009582	detection of abiotic stimulus
GO:0009583	detection of light stimulus
GO:0009584	detection of visible light
GO:0009605	response to external stimulus
GO:0009888	organ development
GO:0014706	striated muscle development
GO:0015020	glucuronosyltransferase activity
GO:0015291	secondary active transmembrane transporter activity
GO:0015629	actin cytoskeleton
GO:0016028	rhabdomere
GO:0016059	deactivation of rhodopsin mediated signaling
GO:0016460	myosin II complex
GO:0016787	hydrolase activity
GO:0016791	phosphoric monoester hydrolase activity
GO:0017171	serine hydrolase activity
GO:0022804	active transmembrane transporter activity
GO:0022857	transmembrane transporter activity
GO:0022892	substrate-specific transporter activity
GO:0030016	myofibril
GO:0030017	sarcomere
GO:0030239	myoblast maturation
GO:0031012	extracellular matrix

GO:0042578	phosphoric ester hydrolase activity
GO:0042592	homeostatic process
GO:0042598	vesicular fraction
GO:0042692	muscle cell differentiation
GO:0043167	ion binding
GO:0043169	cation binding
GO:0043292	contractile fiber
GO:0044420	extracellular matrix
GO:0044421	extracellular region
GO:0044449	contractile fiber
GO:0045445	skeletal muscle fiber development
GO:0046872	metal ion binding
GO:0048469	cell development
GO:0048627	skeletal muscle fiber development
GO:0048628	myoblast maturation
GO:0050906	detection of stimulus during sensory perception
GO:0050908	detection of light stimulus during visual perception
GO:0050953	sensory perception of light stimulus
GO:0050962	detection of light stimulus during sensory perception
GO:0051146	striated muscle cell differentiation
GO:0051606	detection of stimulus
GO:0055001	muscle cell development
GO:0055002	striated muscle cell development
GO:0055074	calcium ion homeostasis

Table S1J: Gene Ontologies Down-regulated in yw, w1118 DR and dSir2 overexpression All gene ontologies have at least 5 genes in the GO and p value < 0.05.

GO:0000070	M phase of mitotic cell cycle
GO:0000074	regulation of progression through cell cycle
GO:0000082	interphase of mitotic cell cycle
GO:0000087	M phase of mitotic cell cycle
GO:0000228	nuclear chromosome
GO:0000278	mitotic cell cycle
GO:0000279	M phase
GO:0000578	embryonic axis specification
GO:0000775	chromosome, pericentric region
GO:0000785	chromatin
GO:0000792	heterochromatin
GO:0000793	condensed chromosome
GO:0000808	origin recognition complex
GO:0000819	sister chromatid segregation
GO:0000910	cytokinesis
GO:0003676	nucleic acid binding
GO:0003677	DNA binding
GO:0003682	chromatin binding
GO:0004672	protein kinase activity
GO:0004674	protein serine/threonine kinase activity
GO:0005515	protein binding
GO:0005622	intracellular
GO:0005623	cell
GO:0005634	nucleus
GO:0005654	nuclear lumen
GO:0005664	nuclear origin of replication recognition complex
GO:0005667	transcription factor complex
GO:0005694	chromosome
GO:0005700	polytene chromosome
GO:0005819	spindle
GO:0005856	cytoskeleton
GO:0006139	nucleobase, nucleoside, nucleotide and nucleic acid metabolic process
GO:0006259	DNA metabolic process

GO:0006260	DNA replication
GO:0006261	DNA-dependent DNA replication
GO:0006281	DNA repair
GO:0006350	transcription
GO:0006351	transcription, DNA-dependent
GO:0006355	regulation of transcription, DNA-dependent
GO:0006357	regulation of transcription from RNA polymerase II promoter
GO:0006366	transcription from RNA polymerase II promoter
GO:0006396	RNA processing
GO:0006464	protein modification process
GO:0006468	protein amino acid phosphorylation
GO:0006793	phosphorus metabolic process
GO:0006796	phosphate metabolic process
GO:0006950	response to stress
GO:0006974	response to DNA damage stimulus
GO:0006996	organelle organization and biogenesis
GO:0007049	cell cycle
GO:0007059	chromosome segregation
GO:0007067	M phase of mitotic cell cycle
GO:0007076	M phase of mitotic cell cycle
GO:0007088	regulation of mitosis
GO:0007143	female meiosis
GO:0007275	multicellular organismal development
GO:0007276	gamete generation
GO:0007292	female gamete generation
GO:0007293	germarium-derived egg chamber formation
GO:0007351	blastoderm segmentation
GO:0008283	cell proliferation
GO:0008356	asymmetric cell division
GO:0008375	acetylglucosaminyltransferase activity
GO:0008595	blastoderm segmentation
GO:0009653	anatomical structure development
GO:0009719	response to endogenous stimulus
GO:0009790	embryonic development
GO:0009880	embryonic development
GO:0009892	negative regulation of metabolic process
GO:0009893	positive regulation of metabolic process
GO:0010467	gene expression
GO:0010468	regulation of gene expression
GO:0015630	microtubule cytoskeleton
GO:0016043	cellular component organization and biogenesis
GO:0016070	RNA metabolic process
GO:0016301	kinase activity
GO:0016310	phosphorylation
GO:0016481	negative regulation of transcription
GO:0016563	transcription activator activity
GO:0016568	chromatin modification
GO:0016572	histone phosphorylation
GO:0016740	transferase activity
GO:0016772	transferase activity, transferring phosphorus-containing groups
GO:0016773	phosphotransferase activity, alcohol group as acceptor
GO:0019219	regulation of nucleobase, nucleoside, nucleotide and nucleic acid metabolic process
GO:0019222	regulation of metabolic process
GO:0019953	sexual reproduction
GO:0022402	cell cycle
GO:0022403	cell cycle phase
GO:0030154	cell differentiation
GO:0030496	midbody
GO:0030727	germarium-derived female germ-line cyst formation
GO:0031323	regulation of cellular metabolic process
GO:0031324	negative regulation of cellular metabolic process
GO:0031325	positive regulation of cellular metabolic process

GO:0031519	PcG protein complex
GO:0032502	developmental process
GO:0032774	RNA biosynthetic process
GO:0032837	distributive segregation
GO:0033301	cell cycle comprising mitosis without cytokinesis
GO:0035102	PRC1 complex
GO:0035186	syncytial blastoderm mitotic cell cycle
GO:0043170	macromolecule metabolic process
GO:0043226	organelle
GO:0043227	membrane-bound organelle
GO:0043228	non-membrane-bound organelle
GO:0043229	intracellular organelle
GO:0043231	intracellular membrane-bound organelle
GO:0043232	intracellular non-membrane-bound organelle
GO:0043234	protein complex
GO:0043283	biopolymer metabolic process
GO:0043412	biopolymer modification
GO:0043687	post-translational protein modification
GO:0044237	cellular metabolic process
GO:0044238	primary metabolic process
GO:0044422	organelle
GO:0044424	intracellular
GO:0044427	chromosome
GO:0044428	nucleus
GO:0044430	cytoskeleton
GO:0044446	intracellular organelle
GO:0044451	nuclear lumen
GO:0044454	nuclear chromosome
GO:0044464	cell
GO:0045448	mitotic cell cycle, embryonic
GO:0045449	regulation of transcription
GO:0045786	negative regulation of progression through cell cycle
GO:0045892	negative regulation of transcription, DNA-dependent
GO:0045935	positive regulation of nucleobase, nucleoside, nucleotide and nucleic acid metabolic process
GO:0045941	positive regulation of transcription
GO:0048134	germ cell development
GO:0048135	female germ-line cyst formation
GO:0048477	oogenesis
GO:0048518	positive regulation of biological process
GO:0048519	negative regulation of biological process
GO:0048522	positive regulation of cellular process
GO:0048523	negative regulation of cellular process
GO:0048646	anatomical structure development
GO:0048856	anatomical structure development
GO:0048869	cellular developmental process
GO:0050789	regulation of biological process
GO:0050794	regulation of cellular process
GO:0051276	chromosome organization and biogenesis
GO:0051301	cell division
GO:0051325	interphase
GO:0051329	interphase of mitotic cell cycle
GO:0051726	regulation of cell cycle
GO:0065007	biological regulation

Table S1K: Gene Ontologies Up-regulated in Dmp53 knockout. All gene ontologies have at least 5 genes in the GO and p value < 0.05.

GO:0004175	endopeptidase activity
GO:0004252	serine-type endopeptidase activity
GO:0006952	defense response
GO:0008233	peptidase activity

GO:0008236	serine-type peptidase activity
GO:0017171	serine hydrolase activity
GO:0050839	cell adhesion molecule binding

Table S1L: Gene Ontologies Down-regulated in Dmp53 knockout. All gene ontologies have at least 5 genes in the GO and p value < 0.05.

GO:0001871	pattern binding
GO:0004553	hydrolase activity, hydrolyzing O-glycosyl compounds
GO:0005214	structural constituent of chitin-based cuticle
GO:0005501	retinoid binding
GO:0005615	extracellular space
GO:0005616	extracellular space
GO:0008010	structural constituent of chitin-based larval cuticle
GO:0008061	chitin binding
GO:0016490	structural constituent of peritrophic membrane
GO:0016491	oxidoreductase activity
GO:0016798	hydrolase activity, acting on glycosyl bonds
GO:0016918	retinal binding
GO:0019840	isoprenoid binding
GO:0019842	vitamin binding
GO:0030246	carbohydrate binding
GO:0030247	polysaccharide binding
GO:0042302	structural constituent of cuticle

Table S1M: Gene Ontologies Up-regulated in yw, w1118 DR, dSir2 overexpression and Dmp53 knockout. All gene ontologies have at least 5 genes in the GO and p value < 0.05. There are no common gene ontologies Down-regulated in yw, w1118 DR, dSir2 overexpression and Dmp53 knockout.

GO:0004175	endopeptidase activity
GO:0004252	serine-type endopeptidase activity
GO:0006952	defense response
GO:0008233	peptidase activity
GO:0008236	serine-type peptidase activity
GO:0017171	serine hydrolase activity

Table S1N: Reproductive-Related Gene Ontologies Down-regulated in yw, w1118 DR. All gene ontologies have at least 5 genes in the GO and p value < 0.05. No Reproductive-Related GOs are Up-Regulated in yw, w1118 DR.

GO:0007126	M phase of meiotic cell cycle
GO:0007127	M phase of meiotic cell cycle
GO:0007131	M phase of meiotic cell cycle
GO:0007143	female meiosis
GO:0007276	gamete generation
GO:0007280	pole cell migration
GO:0007281	germ cell development
GO:0007292	female gamete generation
GO:0007293	germarium-derived egg chamber formation
GO:0007294	germarium-derived oocyte fate determination
GO:0007304	ovarian follicle cell development
GO:0007306	ovarian follicle cell development
GO:0007307	ovarian follicle cell development
GO:0007311	maternal determination of dorsal/ventral axis, oocyte, germ-line encoded
GO:0008354	germ cell migration
GO:0009994	oocyte differentiation
GO:0016321	female meiosis
GO:0019953	sexual reproduction
GO:0030703	eggshell formation

GO:0030706	germarium-derived oocyte differentiation
GO:0030707	ovarian follicle cell development
GO:0030717	karyosome formation
GO:0030720	germarium-derived egg chamber formation
GO:0030727	germarium-derived female germ-line cyst formation
GO:0032837	distributive segregation
GO:0045132	M phase of meiotic cell cycle
GO:0048134	germ cell development
GO:0048135	female germ-line cyst formation
GO:0048477	oogenesis
GO:0051321	meiotic cell cycle
GO:0051327	M phase of meiotic cell cycle

Table S1O: Reproductive-Related Gene Ontologies Down-regulated in Canton-S DR. All gene ontologies have at least 5 genes in the GO and p value < 0.05. No Reproductive-Related GOs are Up-Regulated in Canton-S DR.

GO:0007056	female meiosis
GO:0007126	M phase of meiotic cell cycle
GO:0007127	M phase of meiotic cell cycle
GO:0007131	M phase of meiotic cell cycle
GO:0007143	female meiosis
GO:0007276	gamete generation
GO:0007280	pole cell migration
GO:0007281	germ cell development
GO:0007292	female gamete generation
GO:0007293	germarium-derived egg chamber formation
GO:0007294	germarium-derived oocyte fate determination
GO:0007304	ovarian follicle cell development
GO:0007306	ovarian follicle cell development
GO:0007307	ovarian follicle cell development
GO:0009994	oocyte differentiation
GO:0016321	female meiosis
GO:0019953	sexual reproduction
GO:0030381	ovarian follicle cell development
GO:0030703	eggshell formation
GO:0030706	germarium-derived oocyte differentiation
GO:0030707	ovarian follicle cell development
GO:0030717	karyosome formation
GO:0030720	germarium-derived egg chamber formation
GO:0030727	germarium-derived female germ-line cyst formation
GO:0032837	distributive segregation
GO:0042078	germ cell development
GO:0045132	M phase of meiotic cell cycle
GO:0048134	germ cell development
GO:0048135	female germ-line cyst formation
GO:0048477	oogenesis
GO:0051321	meiotic cell cycle
GO:0051327	M phase of meiotic cell cycle

Table S1P: Reproductive-Related Gene Ontologies Down-regulated in dSir2 Overexpression. All gene ontologies have at least 5 genes in the GO and p value < 0.05. No Reproductive-Related GOs are Up-Regulated in dSir2 Overexpression.

GO:0007143	female meiosis
GO:0007276	gamete generation
GO:0007292	female gamete generation
GO:0007293	germarium-derived egg chamber formation
GO:0019953	sexual reproduction
GO:0030727	germarium-derived female germ-line cyst formation
GO:0032837	distributive segregation

GO:0042078	germ cell development
GO:0048134	germ cell development
GO:0048135	female germ-line cyst formation
GO:0048477	oogenesis
