# Supplementary figures and images for: Comparative transcriptional profiling identifies takeout as a gene that regulates life span
Source: Aging (Albany NY). 2010 May 11;2(5):298–310. doi: 10.18632/aging.100146 (PMC2898020; doi:10.18632/aging.100146)

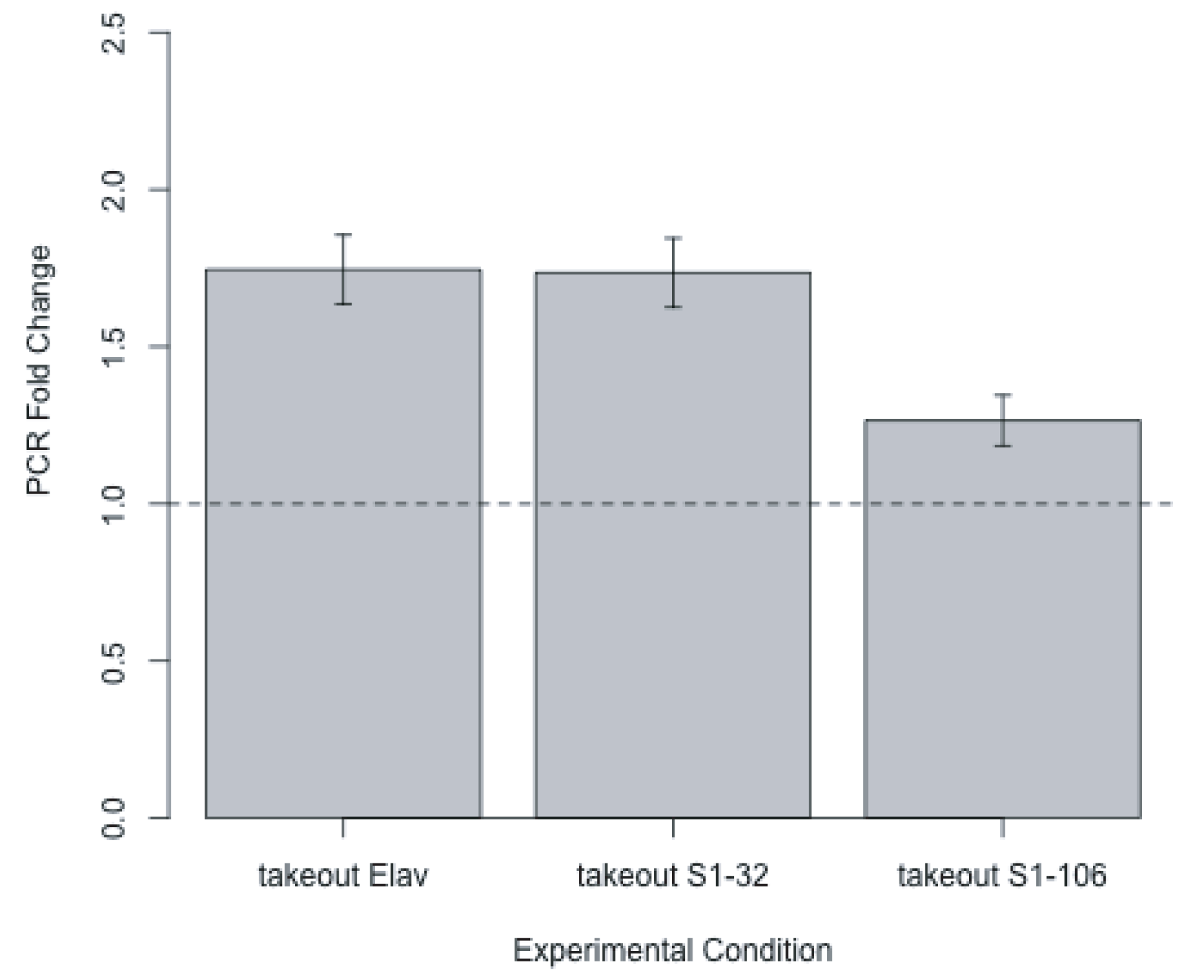

Supplement: Supplementary Figure 2 — takeout mRNA expression is increased in Elav GeneSwitch;UAS-to, S1-32; UAS-to and S1-106; UAS-to. Fold change increase by qPCR of takeout mRNA from 10-Day old flies from these three life span extending conditions as compared to their genetically matched controls. [file aging-02-298-s002.tif]

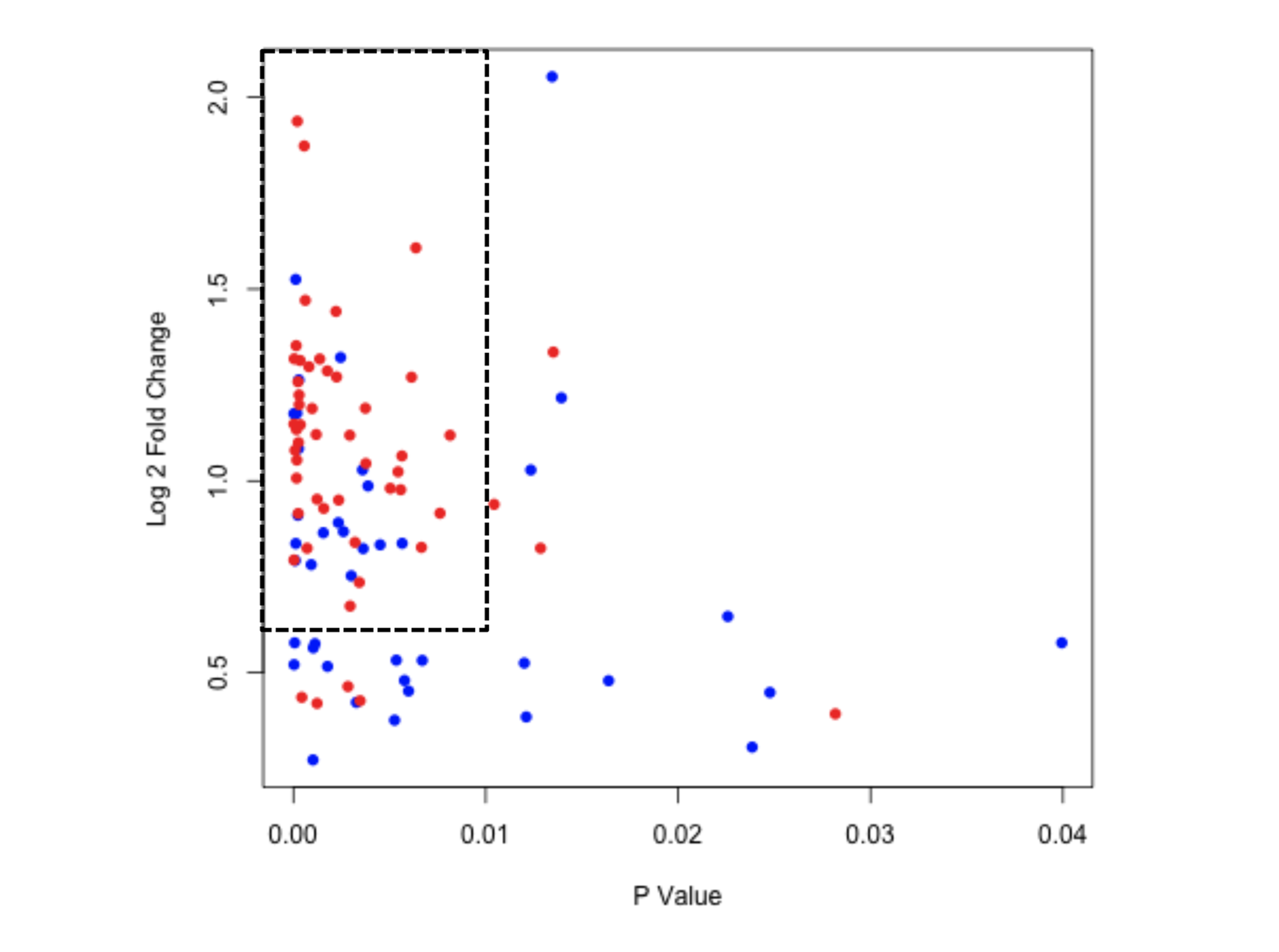

Supplement: Supplementary Figure 3 — Each point on the graph represents a gene measured by both microarray and qPCR. The axes describe the fold change and p value of the microarray data. The red dots represent genes with a significant fold change (>20%) in PCR, and the blue dots represent genes with a non-significant fold change in PCR. The dotted lines define a box of the region where the PCR data is most likely to be significant--fold change > 1.5 (0.58 in log2 space) and p value < 0.01. [file aging-02-298-s003.tif]
